# Enhanced diagnosis of advanced fibrosis and cirrhosis in individuals with NAFLD using FibroScan-based Agile scores

**DOI:** 10.1016/j.jhep.2022.10.034

**Published:** 2022-11-12

**Authors:** Arun J. Sanyal, Julie Foucquier, Zobair M. Younossi, Stephen A. Harrison, Philip N. Newsome, Wah-Kheong Chan, Yusuf Yilmaz, Victor De Ledinghen, Charlotte Costentin, Ming-Hua Zheng, Vincent Wai-Sun Wong, Magdy Elkhashab, Ryan S. Huss, Robert P. Myers, Marine Roux, Aymeric Labourdette, Marie Destro, Céline Fournier-Poizat, Véronique Miette, Laurent Sandrin, Jérôme Boursier

**Affiliations:** 1 Director, Stravitz-Sanyal Institute of Liver Disease and Metabolic Health, VCU School of Medicine and Chair, Division of Gastroenterology, Hepatology and Nutrition in the Department of Internal medicine at VCU School of Medicine, Richmond, VA, USA; 2 Echosens, Paris, France; 3 Inova Fairfax Medical Campus, Falls Church, VA, USA; 4 Radcliffe Department of Medicine, University of Oxford, Oxford, UK; 5 National Institute for Health Research, Birmingham Biomedical Research Centre at University Hospitals Birmingham NHS Foundation Trust, Birmingham, UK & Centre for Liver & Gastrointestinal Research, Institute of Immunology and Immunotherapy, University of Birmingham, Birmingham, UK; 6 Gastroenterology and Hepatology Unit, Department of Medicine, Faculty of Medicine, University of Malaya, Kuala Lumpur, Malaysia; 7 Department of Gastroenterology, School of Medicine, Recep Tayyip Erdogan University, Rize, Turkey; 8 Liver Research Unit, Institute of Gastroenterology, Marmara University, Istanbul, Turkey; 9 Hepatology and Gastroenterology Department, Haut-Lévêque University Hospital, Pessac, France; 10 Hepato-gastroenterology & digestive oncology department, Grenoble-Alpes University Hospital, Grenoble, France; 11 NAFLD Research Center, Department of Hepatology, the First Affiliated Hospital of Wenzhou Medical University, China; 12 Key Laboratory of Diagnosis and Treatment for The Development of Chronic Liver Disease in Zhejiang Province, Wenzhou, China; 13 Department of Medicine and Therapeutics, The Chinese University of Hong Kong, Hong Kong, China; 14 Toronto Liver Centre, Toronto, ON, Canada; 15 Gilead Sciences, Inc., Foster City, CA, USA; 16 The Liver Company, Palo Alto, CA, USA; 17 HIFIH Laboratory, UPRES EA3859, SFR 4208, Angers University, Angers, France; 18 Hepato-Gastroenterology Department, Angers University Hospital, Angers, France

**Keywords:** Vibration-Controlled Transient Elastography, VCTE, Non-invasive test, Non-Alcoholic Fatty Liver Disease, Advanced fibrosis, Cirrhosis

## Abstract

**Background & Aims:**

Currently available non-invasive tests, including fibrosis-4 index (FIB-4) and liver stiffness measurement (LSM by VCTE), are highly effective at excluding advanced fibrosis (AF) (F ≥3) or cirrhosis in people with non-alcoholic fatty liver disease (NAFLD), but only have moderate ability to rule-in these conditions. Our objective was to develop and validate two new scores (Agile 4 and Agile 3+) to identify cirrhosis or AF, respectively, with optimized positive predictive value and fewer indeterminate results, in individuals with NAFLD attending liver clinics.

**Methods:**

This international study included seven adult cohorts with suspected NAFLD who underwent liver biopsy, LSM and blood sampling during routine clinical practice or screening for trials. The population was randomly divided into a training set and an internal validation set, on which the best-fitting logistic regression model was built, and performance and goodness of fit were assessed, respectively. Furthermore, both scores were externally validated on two large cohorts. Cut-offs for high sensitivity and specificity were derived in the training set to rule-out and rule-in cirrhosis or AF and then tested in the validation set and compared to FIB-4 and LSM.

**Results:**

Each score combined LSM, AST/ALT ratio, platelets, sex and diabetes status, as well as age for Agile 3+. Calibration plots for Agile 4 and Agile 3+ indicated satisfactory to excellent goodness of fit. Agile 4 and Agile 3+ outperformed FIB-4 and LSM in terms of AUROC, percentage of patients with indeterminate results and positive predictive value to rule-in cirrhosis or AF.

**Conclusions:**

The two novel non-invasive scores improve identification of cirrhosis or AF among individuals with NAFLD attending liver clinics and reduce the need for liver biopsy in this population.

## Graphical Abstract

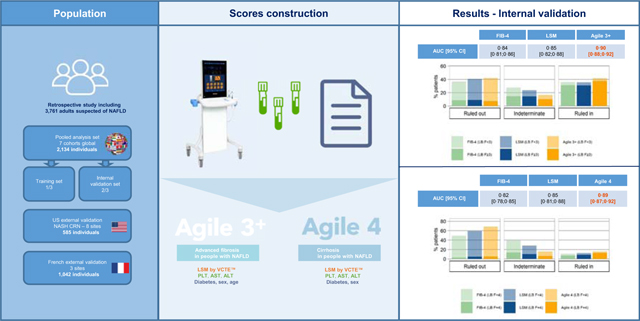

## Introduction

Non-alcoholic fatty liver disease (NAFLD) is a leading cause of liver-related mortality and is already the leading etiology of liver disease requiring liver transplantation in women.^1^ The burden of end-stage liver disease is expected to increase over the coming decade given the high prevalence of NAFLD.^2^ In patients with NAFLD, the fibrosis stage is a critical determinant of prognosis and mortality with a substantial step up in all-cause mortality and liver-related outcomes in those with bridging fibrosis (stage 3 disease) or cirrhosis (stage 4).^3^ These sub-populations are thus at highest risk of outcomes, underscoring the need to identify these individuals within the population with NAFLD.

For patients referred to secondary and tertiary level liver clinics, a key diagnostic objective is to identify those with stage 3 or 4 disease. The current reference standard is histological assessment of liver biopsy (LB) sections. Liver biopsies are invasive and can occasionally cause severe morbidity and even mortality.^4^ Their use is further limited by sampling, intra- and inter-observer variability in interpretation.^5^ These limitations have restricted the widespread use of an LB-based approach in clinical care and serve as a rationale to develop non-invasive tools for this purpose. While a substantial body of literature on the use of laboratory aids such as the FIB-4 score or vibration-controlled transient elastography (VCTE) has been published, none have met regulatory standards for approval and there remains a continued need to develop non-invasive tools to identify those with NAFLD who have AF (F ≥3) or cirrhosis (F = 4).

In this study, we developed and validated two scores (Agile 3+ and Agile 4) to diagnose AF (F ≥3) or cirrhosis (F = 4), respectively, in populations being evaluated for NAFLD. These scores combine liver stiffness measurements (LSM), as measured by VCTE, with additional laboratory and demographic features. These tests have been developed as diagnostic tools to identify patients with a higher probability of having AF or cirrhosis among those being evaluated for NAFLD in secondary and tertiary care hepatology practices. This is expected to inform and assist clinical decision making with respect to initiation of currently recommended standard of care surveillance for hepatocellular cancer and esophageal varices, referral for treatment trials targeting such individuals, and, eventually, for consideration of specific pharmacological treatments when these are established and approved.

The specific goal of this study was to establish the utility of the Agile 3+ and Agile 4 scores for the diagnosis of AF or cirrhosis in those being evaluated for NAFLD in hepatology practices. A secondary goal was to determine if these scores outperformed commonly used approaches such as FIB-4 and LSM measured by VCTE for this purpose. These goals were met by studies with the following objectives: (1) to develop and calibrate the Agile 3+ and 4 scores and establish their sensitivity and specificity for diagnosis of AF or cirrhosis, respectively; (2) to optimize cut-offs to maximize the specificity without clinically relevant loss of sensitivity, to maximize the positive predictive value (PPV) while reducing the proportion of individuals with indeterminant results; (3) to externally validate these findings in independent populations derived from hepatology clinics, *i.e.* the intended use setting; (4) to investigate the impact of BMI, steatosis, diabetes, VCTE probe type and prevalence of the target conditions on performances of the new scores.

## Material and methods

### Description of data

Data from nine cohorts of adult patients who underwent LB for evaluation of NAFLD with concomitant blood work-up for routine biological markers and LSM by VCTE (FibroScan, Echosens, France) were gathered. Data came from North America, Eastern & Western Europe and Asia. The TRIPOD guidelines^6^ were followed to report the development and internal and external validation of the prediction model for diagnosis of cirrhosis and AF (Table S1).

Seven cohorts came from secondary/tertiary hepatology clinics, one cohort came from the baseline visit (including screen failure patients) from a clinical trial and one cohort came from the NAFLD Adult Database 2 of the Non-alcoholic Steatohepatitis Clinical Research Network (NASH CRN, NIDDK) (also all tertiary care hepatology clinics). All cohort data were collected in the framework of a clinical study for which the local ethical committee granted approval and may have already been used completely or in part for other publications (Tables S2 and S3). Patients gave written informed consent to participate in the studies. Each study was conducted in accordance with the Declaration of Helsinki and in agreement with the International Conference on Harmonization guidelines on Good Clinical Practice. FibroScan operators were masked to patients’ clinical and histological data. All LB results were read by expert pathologists blinded by patients’ clinical data and FibroScan results.

Among these nine cohorts, seven were pooled together to constitute the internal dataset that was then randomly split into a training set (TS) and an internal validation set (VS) (2:1) by stratifying on cohort and fibrosis stage. The two other datasets (named “NASH CRN” cohort and “French NAFLD” cohort) were used as external VS. For the French NAFLD cohort, statistical analyses were independently conducted by the investigator (JB) and his team in agreement with all concerned parties.

### Eligibility

Eligible patients were aged 18 years or older and had a LB and a FibroScan examination performed within 6 months. Additionally, a single blood collection with all the required biological parameters was available within 6 months of the LB and 1 month of the FibroScan examination.

Patients who met the following criteria were excluded:

- non-metabolic comorbidities that could have induced liver disease such as viral hepatitis, drug-induced liver injury, excessive alcohol consumption, or HIV;
- less than eight valid measurements for LSM by VCTE;^7^
- missing data for the variables needed in the developed scores and for the fibrosis stage.

Furthermore, in case of patients assessed with both M and XL probes (8.5% of patients from the TS and 8.1% of patients from the internal VS), the FibroScan examination corresponding to the XL probe was only considered when the patients’ BMI was greater or equal to 35 kg/m^2^. In the French NAFLD cohort, the BMI cut-off was 30 kg/m^2 8^. Patients measured with both M and XL probes with missing BMI values were excluded.

### Variables

The main outcomes were the diagnoses of AF (F ≥3) or cirrhosis (F = 4) using the NASH CRN scoring system.^9^ The models considered 16 predictor variables: LSM by VCTE (kPa), age (years), sex, diabetes status (types 1 and 2 regardless of treatment), arterial hypertension (regardless of treatment), BMI (kg/m^2^), aspartate aminotransferase (AST, U/L), alanine aminotransferase (ALT, U/L), AST/ALT ratio (AAR), platelets (PLT, G/L), high-density lipoproteins (mmol/L), low-density lipoproteins (mmol/L), albumin (g/L), gamma glutamyltransferase (U/L), triglycerides (mmol/L), fasting glucose (mmol/L). Those 16 predictors were *a priori* considered to develop the models because they are among the most common and simple routine parameters assessed during the initial evaluation of individuals with NAFLD. Moreover, because of the collinearity between AST and ALT, we performed separate model developments with AST, ALT or AAR. Of these, the model with AAR gave the best discriminative power and was therefore selected.

### Statistical analysis

#### Sample size

The sample size was determined for the development of a clinical prediction model.^10^ To develop a new logistic regression model based on up to 16 candidate predictor parameters and an anticipated Cox-Snell R squared statistic $\left(R_{CS}^{2}\right)$ of at least 0.1, and to target an expected shrinkage factor of 0.9, a sample size of at least 1,358 patients was needed.

#### Construction of the scores

Each of the two scores was developed independently on the TS. The selection of parameters was based on the combination of LSM with clinical parameters and laboratory biomarkers related to liver fibrosis. Each model was developed in three steps:

- Parameters were combined into a multivariable logistic regression model with a backward stepwise selection procedure to select the optimal parameters^11^ (Tables S8 and S9).
- As the obtained models included too many parameters to be easily implemented, simplified models were derived by withdrawing one or several variables (all combinations were tested) from the model obtained at step 1 (full model). The possibility to remove parameters was evaluated using a likelihood ratio test selection procedure on nested models. Simplified models with smaller number of parameters were selected if non-significantly different (*p* ≥0.01) from the full model using the likelihood ratio test (with multiple testing correction)^12^ (Tables S10 and S11).
- Finally, variable transformations were performed using multivariable fractional polynomials^13^ to optimize the models.

#### Overall diagnostic performances

Performances of both the scores were assessed by the goodness of fit, discrimination, and decision curves and compared to LSM alone and FIB-4 used as predictors of the considered target. The goodness of fit (the agreement between observed outcome and prediction) was evaluated using calibration plots^11^ and discrimination using the AUROC. AUROC comparisons were performed using the Delong test (at a two-sided 5% significance level)^14^ using LB fibrosis stage as the reference. To take into account the impact of false positive and false negatives rates, decision curve analysis^15–17^ was also performed (details provided in the supplementary methods).

#### Dual cut-off approach

Optimal rule-out (high sensitivity) and rule-in (high specificity) sets of cut-offs were selected to decrease the number of patients with indeterminate results (in-between the two cut-off values) compared to LSM and FIB-4 and to increase the PPV in the rule-in zone without substantially degrading sensitivity. To do so we tested cut-off values with sensitivity and specificity at 85, 90 and 95% and all their combinations and chose and reported the optimal combinations in the TS. Exactly the same sets of cut-offs were then applied to the VS. Performances when using the usual 90% sensitivity and 90% specificity cut-offs were also reported. Then, for the diagnosis of F4, a rule-in cut-off value with 99% specificity was derived in the TS for FIB-4, LSM and Agile 4 to obtain a very high PPV. When evaluating performance at a given cut-off, sensitivity, specificity, PPV, and negative predictive value (NPV) were computed. At last, for the diagnosis of AF, previously published cut-off values for FIB-4 and LSM^18,19^ were also used for comparison to Agile 3+.

#### Sensitivity analyses

AUROCs of both the scores for patients with BMI ≥30 kg/m^2^ *vs*. BMI <30 kg/m^2^, with steatosis severity S0/S1 vs. S≥2, with vs. without diabetes and with LSM measured with M *vs*. XL probes were compared to evaluate the impact of obesity, steatosis, diabetes, and probe on Agile 4 and Agile 3+.

Since the predictive values depend on the target prevalence, a sensitivity analysis was carried out in order to assess the impact of prevalence on the predictive values at given sensitivity and specificity and therefore at a fixed cut-off. Prevalence of AF varied from 0.05 to 0.55 and that of cirrhosis from 0.02 to 0.25.

Statistical analyses were performed using the R software version 3.6 and subsequent^20^ Packages pROC,^21^ glmnet^22^ and mfp^23^ were used to develop and study the performances of the models.

## Results

### Patient characteristics

The internal dataset consisted of 2,134 patients (flowchart in Fig. S1), of whom 1,434 were in the TS (to construct the scores) and 700 were in the internal VS. As expected, the TS and the internal VS had similar characteristics in terms of collected parameters and distribution of fibrosis stages (Table 1). In both datasets, the prevalence of AF and cirrhosis was 54% and 23%, respectively, which was higher than those expected in patients with NAFLD seen in secondary/tertiary care liver clinics.^24–26^ For external validation, the NASH CRN cohort comprised 585 patients, of whom 13% had cirrhosis and 37% had AF. The French NAFLD cohort comprised 1,042 patients and was very similar to the NASH CRN cohort: 13% had cirrhosis and 38% had AF. Both NASH CRN and French NAFLD cohorts correspond to the intended use population, so for the TS and the internal VS, PPV and NPV were adjusted using a prevalence of 13% for cirrhosis and 37% for AF. As reported in Table 1, the TS and the internal VS had broadly similar demographic, metabolic, and serological characteristics to the external VS. However, while there were as many men as women in the TS (50.8% of men) and in the internal VS (51.3% of men), there were fewer men in the NASH CRN cohort (37.4%) and more men in the French NAFLD cohort (59.7%). Moreover, patients in the French NAFLD cohort had higher ALT values with a median value of 57 U/L in contrast to the TS, internal VS and NASH CRN cohort that had median values ranging from 47 U/L to 49 U/L. Furthermore, as expected, due to the high prevalence of cirrhosis and AF in the TS and in the internal VS, higher values of LSM (~10 kPa in TS and internal VS) were observed compared to LSM in the NASH CRN and the French NAFLD cohorts (~8 kPa). Patient characteristics of each cohort by target are detailed in Tables S4-7.

### Agile 4

#### Score construction

The parameters significantly contributing to the prediction of cirrhosis were LSM, AAR, PLT, sex and diabetes status (details on the predictors selected at each stage of the score construction are presented in Tables S8 and S10). Considering diabetes status: yes = 1, no = 0 and sex: male = 1, female = 0, this resulted in the following equation:

$$Agile\;4=\frac{e^{logit\left(p_{F=4}\right)}}{1+e^{logit\left(p_{F=4}\right)}}$$

with $logit\left(p_{F=4}\right)=7.50139-15.42498\times \frac{1}{\sqrt{LSM}}-0.01378\times PLT-1.41149\times AAR^{-1}-0.53281\times Sex\text{+0.41741}\times Diabetes\;status$

As Agile 4 is the predicted probability of cirrhosis from the logistic regression model, it is bounded between 0 and 1 and can be interpreted in a probabilistic manner.

#### Overall diagnostic performances

On the TS and the internal VS, the calibration line was close to the ideal calibration that conveyed an excellent goodness of fit of predicted probability of cirrhosis (Fig. S2). Furthermore, predictive performances in terms of discrimination of Agile 4 indicated an AUROC of 0.91 (95% CI 0.89–0.92) in the TS and 0.89 (95% CI 0.87–0.92) in the internal VS, significantly different from the AUROC of LSM (Delong test p <0.0001) and FIB-4 (p <0.0001) (Table 2 and Fig. S3). Decision curves (Fig. S4) also suggest that Agile 4 is a better option compared to FIB-4, LSM alone or even treating all patients as having cirrhosis since it has the highest net benefit and the highest clinical value across the range of threshold probabilities (0.0; 0.5).

**Fig. 1.**
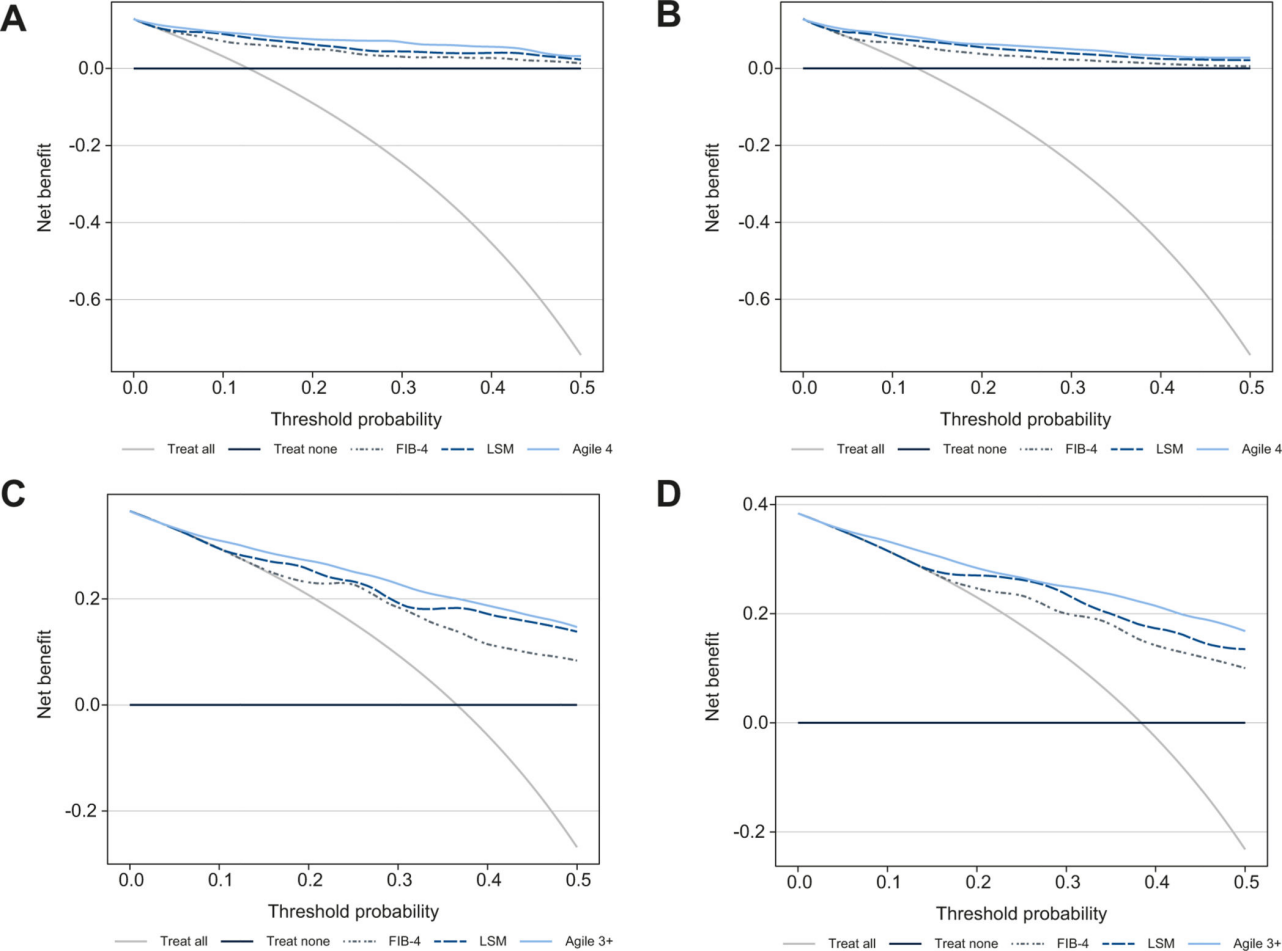
Decision curves of FIB-4, LSM and Agile 4 for the diagnosis of cirrhosis. (A) In the NASH CRN and (B) the French NAFLD cohorts and FIB-4, LSM and Agile 3+ for the diagnosis of advanced fibrosis (C) in the NASH CRN and (D) the French NAFLD cohorts. Decision curves analysis detailed in supplementary methods. FIB-4, fibrosis-4 index; LSM, liver stiffness measurement; NAFLD, non-alcoholic fatty liver disease; NASH CRN, Non-alcoholic Steatohepatitis Clinical Research Network.

Calibration plots were satisfactory for NASH CRN and also for French NAFLD cohorts (Fig. S5 and 6). Though those calibration plots are slightly away from the ideal calibration, most of them fall within the 95% CIs. Excellent discrimination (Table 3) of Agile 4 was observed in both the NASH CRN (AUROC 0.93, 95% CI 0.91–0.96) and the French NAFLD cohorts (AUROC 0.89; 95% CI 0.86–0.92). Moreover, significant differences in the AUROC of Agile 4 were seen compared to that of LSM (*p* <0.0001) in the NASH CRN cohort and that of FIB-4 (*p* = 0.0028 and *p* <0.0001) in both external VSs. Decision curves in the external VSs (Fig. 1A,B) show that, whatever the cohorts and across the range of threshold probabilities (0.0; 0.5), Agile 4 is a better option compared to FIB-4 or even treating all patients as having cirrhosis since it has the highest net benefit. For the NASH CRN cohort, Agile 4 had a higher net benefit than LSM across the range of threshold probabilities between 0.20 and around 0.45. For the French NAFLD cohort, Agile 4 and LSM have similar net benefits.

**Fig. 2.**
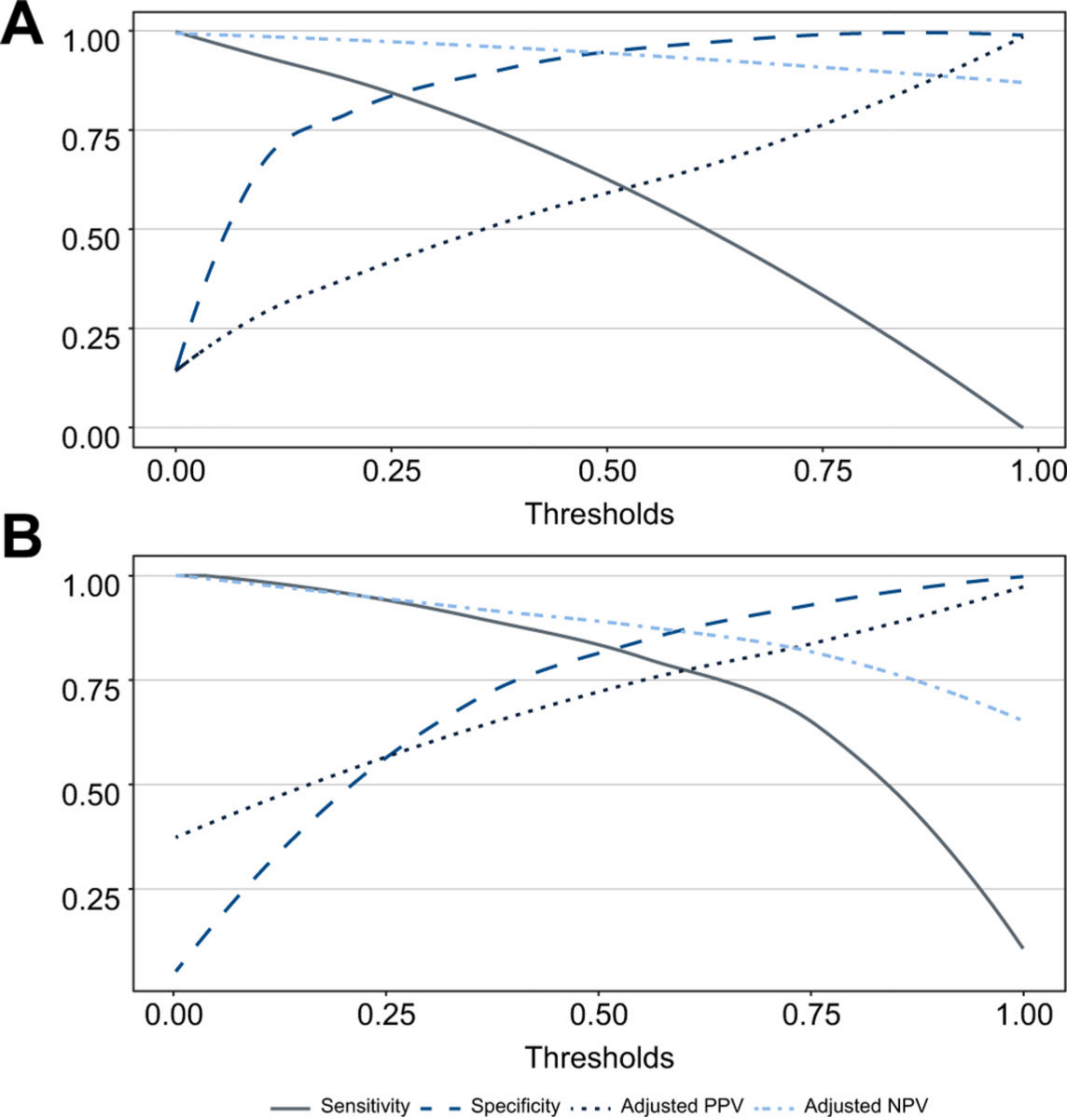
Sensitivity, specificity, adjusted PPV and adjusted NPV of Agile 4 for the diagnosis of cirrhosis and Agile 3+ for the diagnosis of advanced fibrosis in the training set. (A) Agile 4 for the diagnosis of cirrhosis; (B) Agile 3+ for the diagnosis of advanced fibrosis. NPV, negative predictive value; PPV, positive predictive value.

Diagnostic performances of Agile 4 in the TS and the internal VS in terms of sensitivity, specificity, adjusted PPV and NPV are represented in Fig. 2A and Fig. S7, respectively, for all possible cut-off values.

#### Dual cut-off approach

**Fig. 3.**
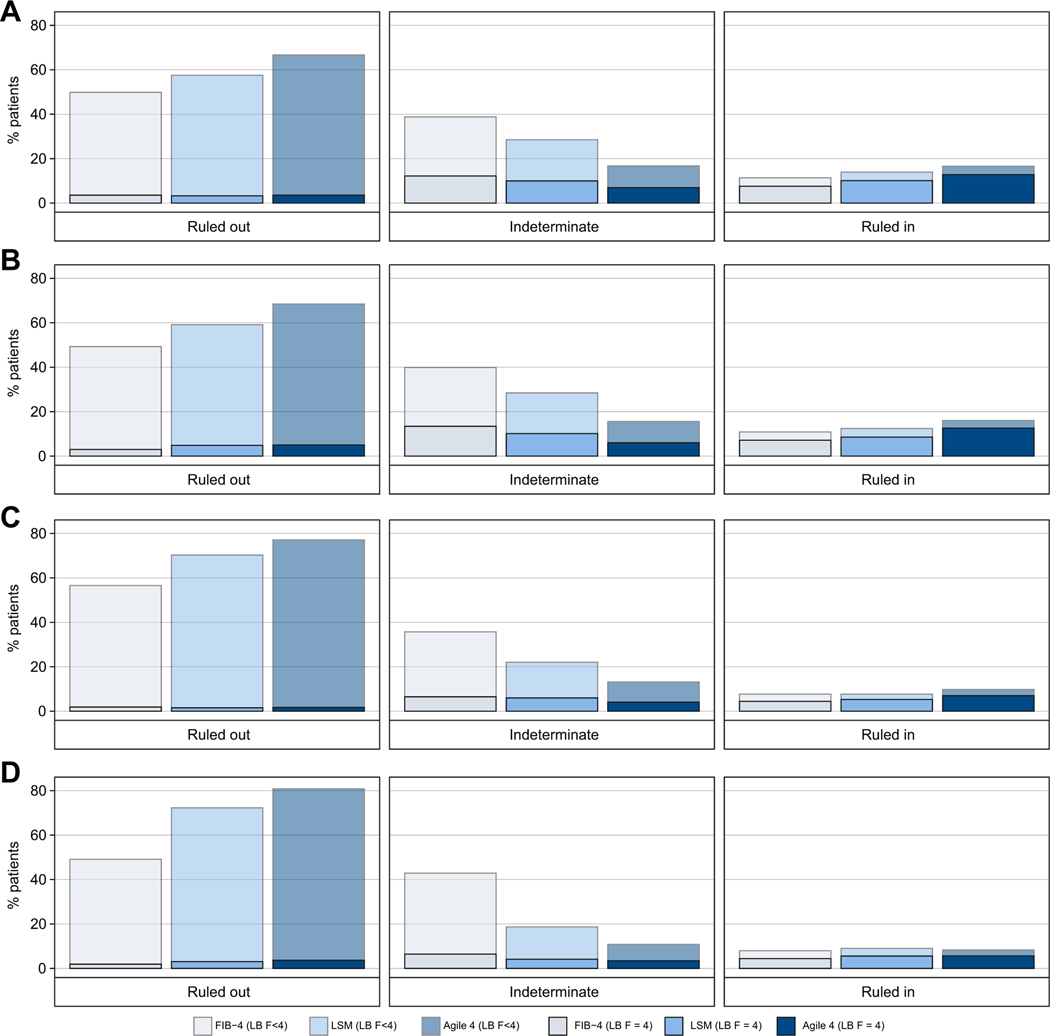
Percentage of patients in rule-out (<85% sensitivity cut-off), indeterminate and rule-in zones (≥90% specificity cut-off) for the diagnosis of cirrhosis with FIB-4, LSM and Agile 4. (A) Training set (n = 1,434), (B) Internal validation set (n = 700), (C) NASH CRN cohort (n = 585), (D) French NAFLD cohort (n = 1,042). On each bar, the solid and transparent parts represent the percentage of patients with and without cirrhosis according to LB, respectively. FIB-4, fibrosis-4 index; LB, liver biopsy; LSM, liver stiffness measurement; NAFLD, non-alcoholic fatty liver disease; NASH CRN, Non-alcoholic Steatohepatitis Clinical Research Network.

To minimize the number of patients in the indeterminate zone and to maximize the PPV in the rule-in zone, it was decided to select a rule-out cut-off that achieved sensitivity of ≥85% and a rule-in cut-off that achieved specificity of ≥95% for the diagnosis of cirrhosis (Table 2). The cut-off values of Agile 4 were 0.251 and 0.565 for rule-out and rule-in, respectively, with characteristics detailed in Table 2, Table 3 and Fig. 3.

Using this approach, no more than 17% of cases had an indeterminate result in the TS and the internal VS. In the TS and the internal VS, an improvement of the proportion of patients correctly/accurately ruled out with high specificities compared to FIB-4 and LSM was observed. Furthermore, the same observation was made in both external VSs. Moreover, the reduction in the numbers of cases with indeterminate results with Agile 4 in all datasets was substantial compared to those achieved using FIB-4 or LSM.

Finally, an improvement in the identification of patients with cirrhosis using Agile 4 was observed. The sensitivity in the rule-in zone was higher than that achieved with FIB-4 or LSM in the TS, the internal VS and the NASH CRN cohort. Moreover, the PPV for Agile 4 increased in all datasets.

Results of the performances of high specificity (99%) cut-off values for the diagnosis of cirrhosis are presented in the supplementary information (Fig. S14, Table S12).

### Agile 3+

#### Score construction

The parameters contributing to the prediction of AF were quite similar to those of Agile 4 as LSM, AAR, PLT, sex and diabetes status remained significant in Agile 3+ as well (details on the predictors selected at each stage of the score construction are presented in Tables S9 and S11). Furthermore, age was also singled out during the construction of Agile 3+.

The equation of Agile 3+ was:

$$Agile\;3+=\frac{e^{logit\left(p_{F\geq 3}\right)}}{1+e^{logit\left(p_{F\geq 3}\right)}}$$

with $logit\left(p_{F\geq 3}\right)=-3.92368+2.29714\times \ln(LSM)-0.00902\times PLT-0.98633\times AAR^{-1}+1.08636\times Diabetes\;status-0.38581\times Sex+0.03018\times Age$

As with Agile 4, Agile 3+ is a predicted probability from the logistic regression model, which is bounded between 0 and 1 and can be interpreted in a probabilistic manner.

#### Overall diagnostic performances

As for Agile 4, for all datasets, the calibration lines of Agile 3+ (Fig. S8–10) were also close to the ideal calibration, which indicates an excellent goodness of fit of predicted probabilities of AF. Excellent discrimination of Agile 3+ was observed with AUROCs around 0.9, significantly different from those of LSM and FIB-4 in the TS, the internal VS and the NASH CRN cohort (Table 2, Table 3 and Fig. S11). Furthermore, decision curves (Fig. 1C,D and Fig. S12) suggest that Agile 3+ is a better option compared to FIB-4, LSM alone (except for the French NAFLD cohort) or even treating all patients as having AF since it has the highest net benefit across the range of threshold probabilities (0.0; 0.5). On the French NAFLD cohort (Fig. 1D), Agile 3+ has the highest net benefit across the range of threshold probabilities between 0.0 and around 0.2 and between about 0.3 and 0.5. For the range between 0.2 and 0.3, Agile 3+ and LSM had similar net benefit that was higher than that of FIB-4.

Diagnostic performances of Agile 3+ in the TS and the internal VS in terms of sensitivity, specificity, adjusted PPV and NPV are represented in Fig. 2B and Fig. S13, respectively, for all possible cut-off values.

#### Dual cut-off approach

**Fig. 4.**
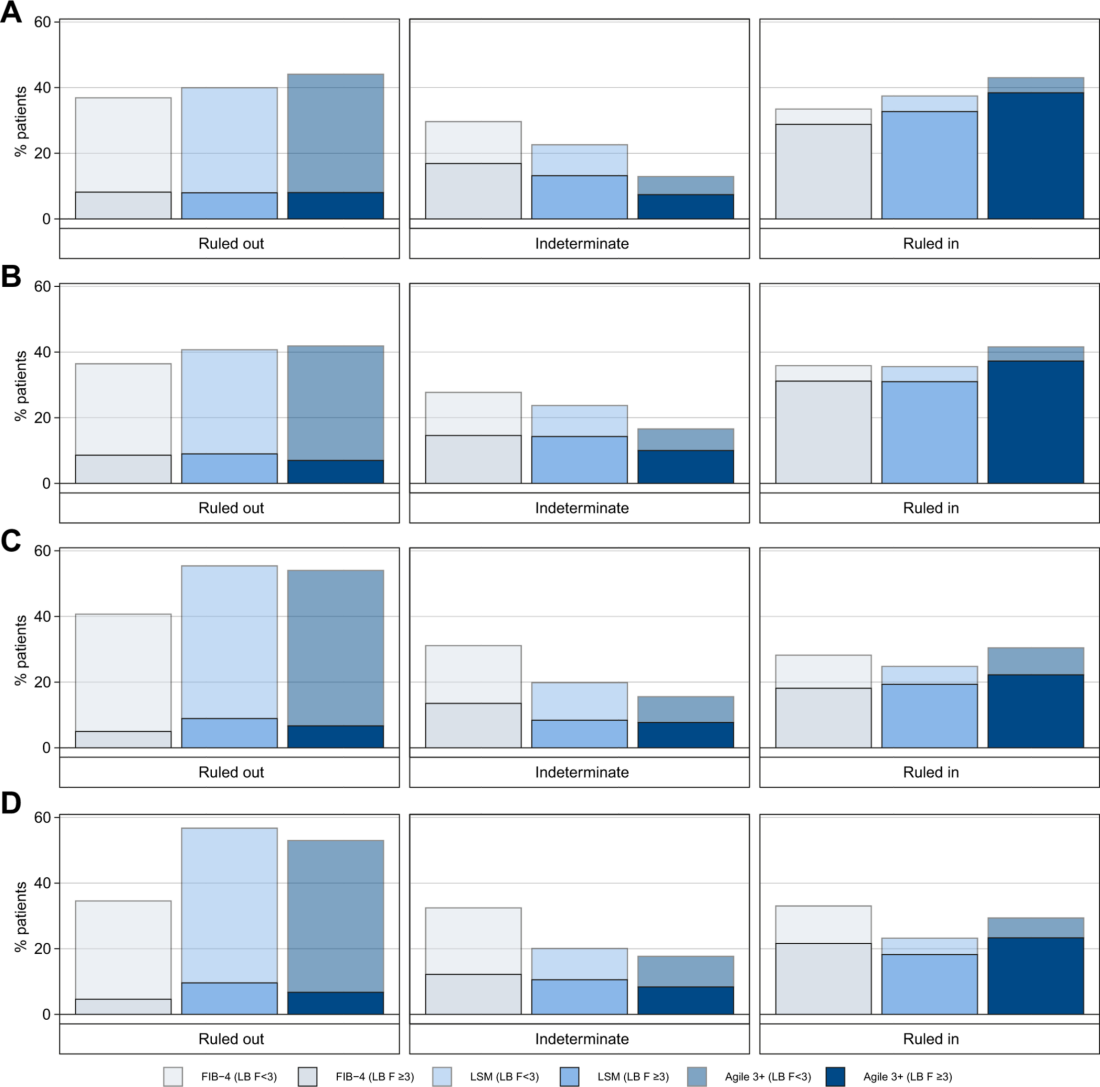
Percentage of patients in rule-out (<85% sensitivity cut-off), indeterminate and rule-in zones (≥90% specificity cut-off) for the diagnosis of advanced fibrosis with FIB-4, LSM and Agile 3+. (A) Training set (n = 1,434), (B) Internal validation set (n = 700), (C) NASH CRN cohort (n = 585), (D) French NAFLD cohort (n = 1,042). On each bar, the solid and transparent parts represent the percentage with and without AF according to LB, respectively. FIB-4, fibrosis-4 index; LB, liver biopsy; LSM, liver stiffness measurement; NAFLD, non-alcoholic fatty liver disease; NASH CRN, Non-alcoholic Steatohepatitis Clinical Research Network.

It was decided to select a rule-out cut-off that achieved sensitivity of ≥85% and a rule-in cut-off that achieved specificity of ≥90% for the diagnosis of F ≥3 (Table 2). Thus, the cut-off values of Agile 3+ were 0.451 and 0.679 for rule-out and rule-in, respectively, characteristics detailed in Table 2, Table 3 and Fig. 4.

No more than 18% of cases had indeterminate results in all datasets with Agile 3+. Moreover, an improvement of the proportion of patients correctly/accurately ruled out with Agile 3+ with high specificities compared to FIB-4 and LSM was observed in the TS and in the internal VS. However, in both external VSs, this increase was confirmed only when comparing Agile 3+ to FIB-4.

Finally, a small improvement of the identification of patients with AF was observed. The sensitivity in the rule-in zone was indeed higher than those of FIB-4 and LSM in all datasets and the PPV slightly increased in the TS and the internal VS. Nevertheless, in both external VSs, even if an improvement of the PPV compared to FIB-4 was observed, the PPV of LSM was higher or equal to that of Agile 3+.

Results of the performances of FIB-4 and LSM using published cut-off values^18,19^ vs. Agile 3+ for the diagnosis of AF are presented in Table S13.

### Sensitivity analyses

**Fig. 5.**
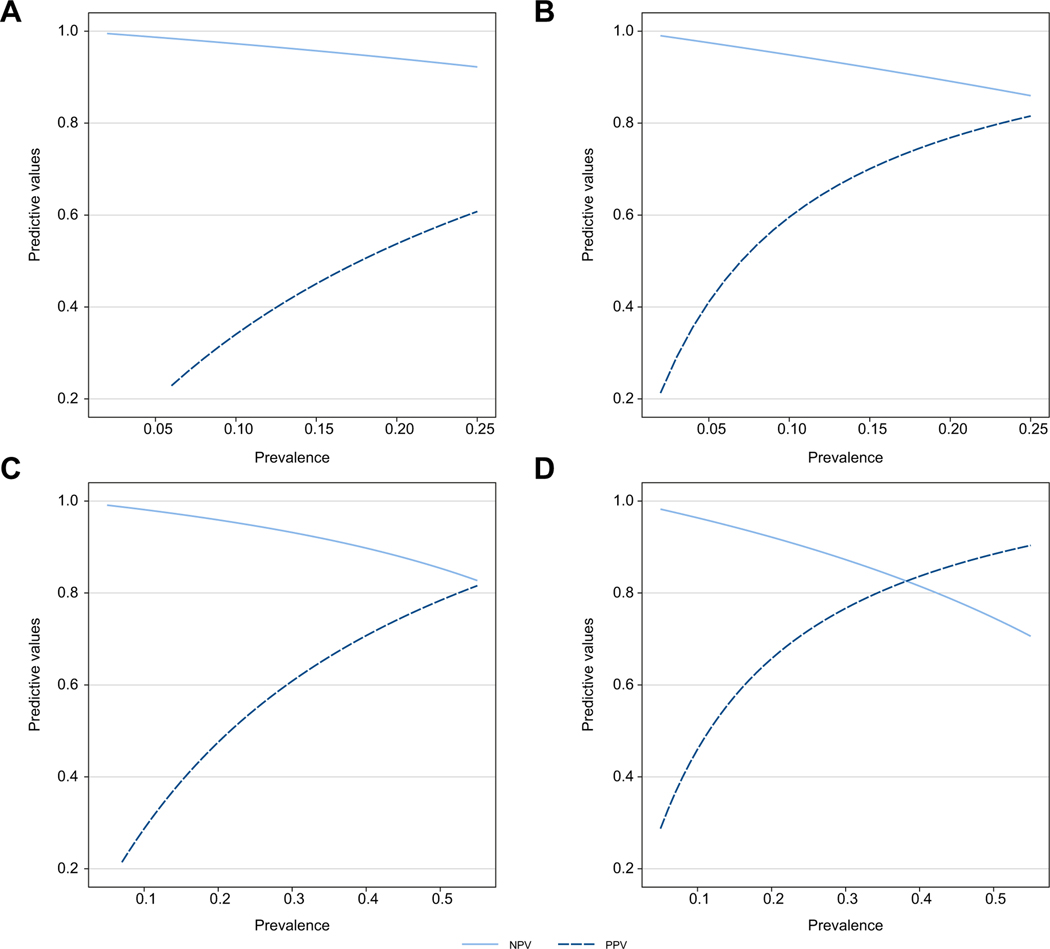
Sensitivity analysis assessing the impact of disease prevalence on PPV and NPV. For Agile 4, the prevalence of cirrhosis was varied from 0.02 to 0.25 for (A) the cut-off of 0.251 (sensitivity = 0.79, specificity = 0.83) and (B) the cut-off of 0.565 (sensitivity = 0.53, specificity = 0.96) to evaluate their impact on NPV and PPV. For Agile 3+, the prevalence of advanced fibrosis was varied from 0.05 to 0.55 for (C) the cut-off of 0.451 (sensitivity = 0.87, specificity = 0.76) and (D) the cut-off of 0.679 (sensitivity = 0.69, specificity = 0.91). NPV, negative predictive value; PPV, positive predictive value.

Results of sensitivity analyses are presented in the supplementary information (Tables S14–17). The AUROCs remain more than 0.80 regardless of whether patients were obese or non-obese, whether they had steatosis or not, whether they had diabetes or not, and whether LSM was measured with an M or XL probe. This demonstrated that these factors do not impact the performances of the models. Finally, impact of the prevalence of AF and cirrhosis on the PPV and NPV for the optimal rule-out and rule-in cut-offs are presented on Fig. 5, for Agile 4 and Agile 3+, respectively. With increasing prevalence of AF and cirrhosis, the PPV tended to increase to a greater extent than the decrease in NPV.

## Discussion

Identifying patients with cirrhosis is of great importance in order to commence periodic surveillance for hepatocellular carcinoma and esophageal varices. Moreover, the identification of patients with AF is also important as these patients are at risk of disease progression towards clinical outcomes. They could benefit in priority from existing interventions and pharmacological therapies for NAFLD once available.

In this study, we propose two new FibroScan-based scores, Agile 4 and Agile 3+, combining LSM with routine biomarkers to identify the presence of cirrhosis or AF, respectively, in secondary/tertiary liver clinics, in patients who would have received a LB for evaluation of NAFLD. By construction, these scores are the probabilities of cirrhosis (Agile 4) and AF (Agile 3+) and can therefore be interpreted as such.

As specified previously, the objectives of this work were to propose new scores and associated sets of rule-out/rule-in cut-offs selected to decrease the number of patients with indeterminate results (in-between the two cut-off values) compared to LSM and FIB-4 and to increase the PPV in the rule-in zone without substantially degrading sensitivity. To do so we tested several levels of sensitivities and specificities. The optimal combinations were rule-out with 85% sensitivity and rule-in with 95% specificity for Agile 4 to predict cirrhosis and rule-out with 85% sensitivity and rule-in with 90% specificity for Agile 3+ to predict AF. Once set on the TS, those same cut-off values were tested in the different VSs and their respective performances confirmed. Nevertheless, performances of both scores using classical rule-out and rule-in cut-off values with 90% sensitivity and 90% specificity, respectively, are presented in Table S18.

This study has the following strengths. Firstly, the scores were derived from a large cohort of 1,434 patients recruited in secondary/tertiary liver clinics from North America, Europe and Asia. Secondly, the study was able to validate the scores in three other large cohorts: (i) an internal VS made from the remaining third of the initial global pool of patients not used for the TS, (ii) a large subset of patients from the NAFLD Adult Database 2 of the NASH CRN conducted in eight expert centers in the USA and (iii) a large cohort of patients from three expert centers in France. This contributed to limit the overfitting. Moreover, the shrinkage factor used to determine the sample size was *a priori* defined at 0.9 (close to 1), high enough to minimize potential model overfitting. Thirdly, these scores were developed using widely available routine biomarkers. By doing so and making the score formula public and available through an app and a website, we aim to make the scores easily and readily accessible without additional cost, at the same time as LSM by VCTE is obtained. Nevertheless, we also compared in all the datasets, the performances of two scenarios: (i) Agile scores calculated for all patients, or (ii) patients first undergo LSM by VCTE then Agile is performed only on patients who are either ruled-in or indeterminate with LSM (Figs S15–17). The results show that, compared to Agile scores alone, sequential use of LSM followed by Agile scores slightly increases the number of patients ruled out and slightly decreases the number of cases with indeterminate results while improving PPV.

However, there are some limitations to this study. LSM by VCTE, for which access is limited across the globe, is needed for the computation of the scores. However, these scores are intended to be used in secondary/tertiary liver clinics where most of the 7,800+ FibroScan devices are currently based. Moreover, the cost of the procedure is covered by public and/or private health care insurance in many countries. Another potential limitation could be the higher prevalence of AF and cirrhosis in the TS and the internal VS compared to the one expected in the intended use population and observed in the external VS. First, to avoid optimistic bias, predictive values reported for the TS and the internal VS were adjusted to the prevalence of the context of use population (namely, the prevalence of the external VSs). Second, the impact of lower prevalence of the target conditions on the predictive values for the selected cut-off values (Fig. 5) was evaluated. With increasing prevalence of AF and cirrhosis, the PPV tended to increase to a greater extent than the decrease in NPV. This means that the cut-off values proposed here would have to be adjusted and the scores need further evaluation in context of use with lower target prevalence. Notwithstanding, it should be noted that developing the score on a TS with a high prevalence of the target conditions allowed us to capture more variability. Another limitation could be the selection and misclassification biases associated with the use of patients who underwent a LB. Therefore, the next step, to further assess the added value of these scores independent from LB, would be to investigate their capacity to predict clinical outcome.

Another limitation is the use of LB as reference standard. First, it is now well recognized that there is a significant intra- and inter-observer variability for the assessment of a fibrosis lesion. One could argue that all LB from the different cohorts should have been assessed centrally by several pathologists with a consensus. However, we believe that by using fibrosis stage assessed by different pathologist(s), expert in the field of chronic liver diseases, the resulting scores should be more robust and independent of the pathologist reading and thus more translatable to real world practice. Moreover, the inter-observer agreement for fibrosis stage has been shown to be excellent.^9,27^ Second, biomarkers used in the scores may have been used to decide on performing the biopsy. However, since the scores are built using routine biomarkers, it is difficult to avoid this selection bias, and the fact that the criteria used by the investigators to request a LB were not homogeneous among the different cohorts may have decreased this potential bias. Third, no criteria concerning the quality of LB was required to be included in this study. However, the comparisons of AUROCs of Agile 3+ and Agile 4 for patients with LB length >15 mm vs. LB length ≤15 mm presented in Table S19 demonstrate that performances were not significantly different between subgroups. Together, these data demonstrate that the performance metrics of the scores were not adversely impacted by the biopsy length and support the robustness of the models.

Finally, it has been shown, for existing scores, that the use of age as one of the markers, as it is the case for Agile 3+, may warrant the use of age-adjusted cut-off values.^18^ Similarly, use of presence of comorbidities such as presence of diabetes can impact the performance of the scores when used in populations with lower or higher prevalence of diabetes (such as in endocrinology).^28^ Therefore, these points need to be further investigated.

In conclusion, by combining simple clinical parameters together with routine laboratory biomarkers and LSM by VCTE, it is possible to identify cirrhosis and AF with improved PPV and fewer indeterminate results in individuals with NAFLD, in secondary/tertiary care liver clinics where the prevalence is at least 13% and 37%, respectively. The use of these non-invasive scores would reduce the need for confirmatory LB, thus improving patient care and reducing associated costs. Agile 4 could also be of interest to adjust pharmacological treatment regimens in case of the presence of cirrhosis. The potential serial use of Agile 3+ and Agile 4 to monitor disease progression or their use to predict clinical outcome needs to be investigated.

## Supplementary Material

1

2

3

## Figures and Tables

**Table 1.** Training set, internal validation set, NASH CRN cohort and French NAFLD cohort patient characteristics.

	Training set n = 1,434		Internal VS n = 700		NASH CRN cohort n = 585		French NAFLD cohort* n = 1,042	
	Median (IQR) or n (%)	N	Median (IQR) or n (%)	N	Median (IQR) or n (%)	N	Median (IQR) or n (%)	N
**Demographics**								
Age (years)	55.0 (16.0)	1,434	55.5 (16.0)	700	54.0 (17.0)	585	58.0 (15.4)	1,042
Male sex	729 (50.8%)	1,434	359 (51.3%)	700	219 (37.4%)	585	622 (59.7%)	1,042
BMI (kg/m^2^)	31.7 (7.80)	1,325	31.6 (8.05)	646	34.6 (9.10)	584	31.2 (7.7)	1,037

**Metabolic**								
Diabetes (type 1 and 2)	723 (50.4%)	1,434	357 (51.0%)	700	268 (45.8%)	585	508 (48.8%)	1,042
Hypertension	719 (50.1%)	1,434	344 (49.1%)	700	334 (57.1%)	585	–	–**

**Blood**								
AST (U/L)	39.0 (31.0)	1,434	38.0 (29.0)	700	37.0 (28.0)	585	39.5 (26.0)	1,042
ALT (U/L)	49.0 (47.0)	1,434	47.0 (45.2)	700	48.0 (42.0)	585	57.0 (45.0)	1,042
AAR	0.808 (0.381)	1,434	0.820 (0.397)	700	0.793 (0.332)	585	0.72 (0.37)	1,042
Platelets count (G/L)	219 (94.0)	1,434	222 (95.2)	700	228 (92.0)	585	218 (85.0)	1,042
HDL (mmol/L)	1.14 (0.414)	1,114	1.11 (0.390)	541	1.11 (0.388)	581	1.14 (0.390)	997
LDL (mmol/L)	2.59 (1.32)	1,088	2.56 (1.24)	530	2.61 (1.33)	568	–	–***
Albumin (g/L)	45.0 (5.00)	1,338	44.0 (5.00)	654	44.0 (4.00)	583	43.0 (5.0)	1,033
GGT (IU/L)	58.0 (70.0)	1,337	61.0 (71.8)	654	43.0 (53.0)	581	77.5 (106.3)	1,042
Triglycerides (mmol/L)	1.69 (1.04)	1,119	1.64 (1.03)	545	1.62 (1.11)	581	1.53 (1.03)	1,002
Fasting glucose (mmol/L)	6.11 (2.36)	1,315	6.10 (2.39)	645	5.88 (1.94)	582	5.80 (2.30)	1,011

**Non-invasive tests**								
FIB-4	1.40 (1.25)	1,434	1.40 (1.20)	700	1.30 (1.06)	585	1.40 (1.17)	1,042
LSM by VCTE (kPa)	10.8 (10.2)	1,434	10.5 (9.83)	700	8.60 (7.20)	585	8.50 (6.70)	1,042

**Fibrosis stage**								
NASH CRN scoring system		1,434		700		585		1,042
F0	202 (14.1%)		97 (13.9%)		121 (20.7%)		116(11.1%)	
F1	269 (18.8%)		130 (18.6%)		134 (22.9%)		240 (23.0%)	
F2	191 (13.3%)		93 (13.3%)		116 (19.8%)		286 (27.5%)	
F3	437 (30.5%)		215 (30.7%)		139 (23.8%)		267 (25.6%)	
F4	335 (23.4%)		165 (23.6%)		75 (12.8%)		133 (12.8%)	

AAR, AST/ALT ratio; ALT, alanine aminotransferase; AST, aspartate aminotransferase; FIB-4, Fibrosis-4 index; GGT, gamma-glutamyltransferase; HDL, high-density lipoprotein; LDL, low-density lipoprotein; LSM, liver stiffness measurement; NAFLD, non-alcoholic fatty liver disease; NASH CRN, Non-alcoholic Steatohepatitis Clinical Research Network; VCTE, vibration-controlled transient elastography; VS, validation set.

* Analysis performed by Professor Boursier and his team.

** 22% missing data.

*** 51% missing data. Results are median (IQR) and number of available data for numeric parameters and n (%) for categorical parameters.

**Table 2.** Diagnostic performances of FIB-4, LSM and Agile 3+ for the diagnosis of advanced fibrosis and of FIB-4, LSM and Agile 4 for the diagnosis of cirrhosis in the training and internal validation sets.

	Training set			Internal validation set		
	FIB-4	LSM	Agile	FIB-4	LSM	Agile
**F4 target**						
AUROC [95% CI]	0.83	0.86	0.91	0.82	0.85	0.89
	[0.80–0.85]	[0.84–0.89]	[0.89–0.92]	[0.78–0.85]	[0.81–0.88]	[0.87–0.92]
Delong test *p* (vs. Agile 4)	<0.0001	<0.0001	n.a.	<0.0001	<0.0001	n.a.
*Rule-out cut-off* (≥*85% Se*)	*<1.39*	*<12.1*	*<0.251*	*<1.39*	*<12.1*	*<0.251*
% patients	50%	58%	67%	49%	59%	68%
Se [95% CI]/Sp [95% CI]	0.85	0.86	0.85	0.87	0.79	0.79
	[0.81–0.89]/0.60	[0.82–0.90]/0.71	[0.81–0.89]/0.82	[0.82–0.92]/0.61	[0.73–0.85]/0.71	[0.72–0.85]/0.83
	[0.57–0.63]	[0.68–0.74]	[0.80–0.84]	[0.57–0.65]	[0.67–0.75]	[0.80–0.86]
NPV	0.96*	0.97*	0.97*	0.97*	0.96*	0.96*
*Indeterminate zone (85% Se; 95% Sp)*						
% patients	39%	29%	17%	40%	28%	16%
*Rule-in cut-off* (≥95% *Sp*)	*≥3.25*	*≥23.2*	*≥0.565*	*≥3.25*	*≥23.2*	*≥0.565*
% patients	11%	14%	17%	11%	12%	16%
Se [95% CI]/Sp [95% CI]	0.33	0.43	0.55	0.30	0.36	0.53
	[0.28–0.38]/0.95	[0.38–0.48]/0.95	[0.50–0.60]/0.95	[0.23–0.37]/0.95	[0.29–0.43]/0.95	[0.45–0.61]/0.96
	[0.93–0.96]	[0.94–0.96]	[0.94–0.96]	[0.93–0.97]	[0.93–0.97]	[0.94–0.98]
PPV	0.50*	0.56*	0.63*	0.48*	0.52*	0.64*

**F ≥3 target**						
AUROC [95% CI]	0.82	0.86	0.90	0.84	0.85	0.90
	[0.80–0.84]	[0.84–0.88]	[0.88–0.91]	[0.81–0.86]	[0.82–0.88]	[0.88–0.92]
Delong test *p* (vs. Agile 3+)	<0.0001	<0.0001	n.a.	<0.0001	<0.0001	n.a.
*Rule-out cut-off* (≥*85% Se*)	*<1.12*	*<9.2*	*<0.451*	*<1.12*	*<9.2*	*<0.451*
% patients	37%	40%	44%	36%	41%	42%
Se [95% CI]/Sp [95% CI]	0.85	0.85	0.85	0.84	0.83	0.87
	[0.82–0.88]/0.62	[0.82–0.88]/0.69	[0.82–0.88]/0.78	[0.80–0.88]/0.61	[0.79–0.87]/0.69	[0.84–0.90]/0.76
	[0.58–0.66]	[0.65–0.73]	[0.75–0.81]	[0.56–0.66]	[0.64–0.74]	[0.71–0.81]
NPV	0.87*	0.89*	0.90*	0.87*	0.88*	0.91*
*Indeterminate zone* (*85% Se; 90% Sp*)						
% patients	30%	23%	13%	28%	24%	17%
*Rule-in cut-off* (≥*90% Sp*)	*≥1.81*	*≥13.6*	*≥0.679*	*≥1.81*	*≥13.6*	*≥0.679*
% patients	33%	37%	43%	36%	36%	42%
Se [95% CI]/Sp [95% CI]	0.53	0.61	0.71	0.57	0.57	0.69
	[0.49–0.57]/0.90	[0.58–0.64]/0.90	[0.68–0.74]/0.90	[0.52–0.62]/0.90	[0.52–0.62]/0.90	[0.64–0.74]/0.91
	[0.88–0.92]	[0.88–0.92]	[0.88–0.92]	[0.87–0.93]	[0.87–0.93]	[0.88–0.94]
PPV	0.76*	0.78*	0.81*	0.77*	0.77*	0.81*

FIB-4, fibrosis-4 index; LSM, liver stiffness measurement; NAFLD, non-alcoholic fatty liver disease; NPV, negative predictive value; PPV, positive predictive value; Se, sensitivity; Sp, specificity. AUROC comparisons were performed using the Delong test (at a two-sided 5% significance level).

* Due to the high prevalence of cirrhosis and AF in the TS and the internal VS, PPV and NPV for these datasets were adjusted on the prevalence of external validation, i.e. F4 = 13% and F ≥3 = 37%.

**Table 3.** Diagnostic performances of FIB-4, LSM and Agile 3+ for the diagnosis of advanced fibrosis and of FIB-4, LSM and Agile 4 for the diagnosis of cirrhosis in the external validation sets.

	NASH CRN cohort			French NAFLD cohort*		
	FIB-4	LSM	Agile	FIB-4	LSM	Agile
**F4 target**						
AUROC [95% CI]	0.83	0.89	0.93	0.81	0.88	0.89
	[0.79–0.88]	[0.86–0.93]	[0.91–0.96]	[0.77–0.85]	[0.85–0.91]	[0.86–0.92]
Delong test *p* (vs. Agile 4)	<0.0001	0.0028	n.a.	<0.0001	0.2363	n.a.
*Rule-out cut-off* (≥*85% Se*)	*<1.39*	*<12.1*	*<0.251*	*<1.39*	*<12.1*	*<0.251*
% patients	57%	70%	77%	49%	72%	81%
Se [95% CI]/Sp [95% CI]	0.85	0.88	0.87	0.85	0.76	0.71
	[0.77–0.93]/0.63	[0.81–0.95]/0.79	[0.79–0.95]/0.86	[0.82–0.881]/0.54	[0.73–0.79]/0.79	[0.68–0.74]/0.88
	[0.59–0.67]	[0.75–0.83]	[0.83–0.89]	[0.50–0.58]	[0.76–0.82]	[0.86–0.90]
NPV	0.97	0.98	0.98	0.96	0.96	0.96
*Indeterminate zone* (*85% Se; 95% Sp*)						
% patients	36%	22%	13%	43%	19%	11%
*Rule-in cut-off* (≥*95% Sp*)	*≥3.25*	*≥23.2*	*≥0.565*	*≥3.25*	*≥23.2*	*≥0.565*
% patients	8%	8%	10%	8%	9%	8%
Se [95% CI]/Sp [95% CI]	0.35	0.41	0.55	0.35	0.44	0.44
	[0.24–0.46]/0.96	[0.30–0.52]/0.97	[0.44–0.66]/0.97	[0.25–0.45]/0.96	[0.34–0.54]/0.96	[0.33–0.55]/0.97
	[0.94–0.98]	[0.96–0.98]	[0.96–0.98]	[0.92–1.00]	[0.92–1.00]	[0.93–1.00]
PPV	0.58	0.69	0.72	0.55	0.62	0.68

**F ≥3 target**						
AUROC [95% CI]	0.78	0.83	0.86	0.78	0.84	0.87
	[0.74–0.82]	[0.80–0.87]	[0.84–0.89]	[0.76–0.81]	[0.81–0.86]	[0.85–0.89]
Delong test *p* (vs. Agile 3+)	<0.0001	0.0042	n.a.	<0.0001	0.0011	n.a.
*Rule-out cut-off* (≥*85% Se*)	*<1.12*	*<9.2*	*<0.451*	*<1.12*	*<9.2*	*<0.451*
% patients	41%	55%	54%	35%	57%	53%
Se [95% CI]/Sp [95% CI]	0.86	0.76	0.82	0.88	0.75	0.83
	[0.81–0.91]/0.56	[0.70–0.82]/0.73	[0.77–0.87]/0.75	[0.85–0.91]/0.49	[0.72–0.78]/0.77	[0.80–0.86]/0.75
	[0.51–0.61]	[0.68–0.78]	[0.71–0.79]	[0.44–0.54]	[0.74–0.80]	[0.71–0.79]
NPV	0.88	0.84	0.88	0.87	0.83	0.87
*Indeterminate zone* (*85% Se; 90% Sp*)						
% patients	31%	20%	16%	32%	20%	18%
*Rule-in cut-off* (≥*90% Sp*)	*≥1.81*	*≥13.6*	*≥0.679*	*≥1.81*	*≥13.6*	*≥0.679*
% patients	28%	25%	30%	33%	23%	29%
Se [95% CI]/Sp [95% CI]	0.50	0.53	0.61	0.56	0.48	0.61
	[0.43–0.57]/0.84	[0.46–0.60]/0.91	[0.54–0.68]/0.87	[0.51–0.61]/0.82	[0.42–0.54]/0.92	[0.55–0.67]/0.90
	[0.80–0.88]	[0.88–0.94]	[0.34–0.90]	[0.78–0.86]	[0.89–0.95]	[0.87–0.93]
PPV	0.64	0.78	0.73	0.65	0.79	0.79

FIB-4, fibrosis-4 index; LSM, liver stiffness measurement; NAFLD, non-alcoholic fatty liver disease; NPV, negative predictive value; PPV, positive predictive value; Se, sensitivity; Sp, specificity. AUROC comparisons were performed using the Delong test (at a two-sided 5% significance level).

* Analysis performed by Professor Boursier and his team.

## Data Availability

Data used for this work are unavailable to access because they are confidential.

## Highlights

- Non-invasive tests’ ability to rule-in advanced fibrosis and cirrhosis is moderate.
- Consequently, two new FibroScan-based scores are proposed: Agile 3+ and Agile 4.
- They demonstrate fewer indeterminate results and higher positive predictive value.
- Clinical performances are globally validated in two large independent cohorts.
- Use of these scores in clinical practice could reduce the need for liver biopsy.

## Impact and implications

Non-invasive tests currently used to identify patients with advanced fibrosis or cirrhosis, such as fibrosis-4 index and liver stiffness measurement by vibration-controlled transient elastography, have high negative predictive values but high false positive rates, while results are indeterminate for a large number of cases. This study provides scores that will help the clinician diagnose advanced fibrosis or cirrhosis. These new easy-to-implement scores will help liver specialists to better identify (1) patients who need more intensive follow-up, (2) patients who should be referred for inclusion in therapeutic trials, and (3) which patients should be treated with pharmacological agents when effective therapies are approved.

## Abbreviations

AAR: AST/ALT ratio
AF: advanced fibrosis
ALT: alanine aminotransferase
AST: aspartate aminotransferase
FIB-4: fibrosis-4
LB: liver biopsy
LSM: liver stiffness measurement
NAFLD: non-alcoholic fatty liver disease
NASH-CRN: Nonalcoholic Steatohepatitis Clinical Research Network
NPV: negative predictive value
PPV: positive predictive value
Se: sensitivity
Sp: specificity
TS: training set
VCTE: vibration-controlled transient elastography
VS: validation set
