# Supplementary material for: Enhanced diagnosis of advanced fibrosis and cirrhosis in individuals with NAFLD using FibroScan-based Agile scores
Source: J Hepatol. Author manuscript; Available in PMC 2023 May 10. (PMC10170177; doi:10.1016/j.jhep.2022.10.034)
Supplement: 1 [file NIHMS1884485-supplement-1.pdf]

Journal of Hepatology, Volume *78*

Supplemental information

Enhanced diagnosis of advanced fibrosis and cirrhosis in individuals with NAFLD using FibroScan-based Agile scores

Arun J. Sanyal, Julie Foucquier, Zobair M. Younossi, Stephen A. Harrison, Philip N. Newsome, Wah-Kheong Chan, Yusuf Yilmaz, Victor De Ledinghen, Charlotte Costentin, Ming-Hua Zheng, Vincent Wai-Sun Wong, Magdy Elkhashab, Ryan S. Huss, Robert P. Myers, Marine Roux, Aymeric Labourdette, Marie Destro, Céline Fournier-Poizat, Véronique Miette, Laurent Sandrin, and Jérôme Boursier

Enhanced diagnosis of advanced fibrosis and cirrhosis in individuals with NAFLD using FibroScan-based Agile scores

Arun J. Sanyal, Julie Foucquier, Zobair M. Younossi, Stephen A. Harrison, Philip N. Newsome, Wah-Kheong Chan, Yusuf Yilmaz, Victor De Ledinghen, Charlotte Costentin, Ming-Hua Zheng, Vincent Wai-Sun Wong, Magdy Elkhashab, Ryan S. Huss, Robert P. Myers, Marine Roux, Aymeric Labourdette, Marie Destro, Céline Fournier-Poizat, Véronique Miette, Laurent Sandrin, Jérôme Boursier

Table of contents

Supplementary methods....2

Supplementary figures....7

Supplementary tables....23

Supplementary references....51

Supplementary methods

Decision curve analysis

Decision curve analyses [1–3] were performed by calculating a clinical net benefit for FIB-4, LSM, Agile 3+ for the diagnosis of AF and FIB-4, LSM and Agile 4 for the diagnosis of cirrhosis in comparison to default strategies of treating all patients as having ("Treat all") or not having ("Treat none") AF or cirrhosis respectively. The net benefit put benefits and harms on the same scale so that they can be compared directly. It is calculated across a range of threshold probabilities (ThresP) considering the number of incorrect diagnoses a clinician is willing to accept to find one correct diagnosis of cirrhosis or AF using Agile 4, Agile 3+, FIB-4 and LSM instead of a liver biopsy (for example, if a clinician found it acceptable to use Agile 4 on 11 patients to find one correct diagnosis of cirrhosis, then the threshold probability is 1 correct diagnosis / 10 incorrect diagnoses = 10%) as:

$$Net\ benefit = \frac{TP}{N} - \frac{FP}{N} \times \frac{ThresP}{1 - \ ThresP}$$

Where TP is the true positive count, FP is the false positive count and N is the total number of patients. The strategy with the highest net benefit at a particular threshold probability has the highest clinical value.

Adjusted predictive values

The relationship between predictive values and the prevalence used for adjustment and sensitivity analysis was:

$$PPV = \frac{Se \times prevalence}{Se \times prevalence + \ (1 - Sp) \times (1 - prevalence)}$$

$$NPV = \frac{Sp \times (1 - prevalence)}{(1 - Se) \times prevalence + Sp \times (1 - prevalence)}$$

Performances of high specificity cut-off values for the diagnosis of cirrhosis

Rule-in cut-off values for at least 99% of specificity for the diagnosis of cirrhosis were derived in the training set for FIB-4, LSM and Agile 4. The rule-in cut-off of Agile 4 was then 0.843 with characteristics detailed in Table S12 and the dual cut-off approach represented in Fig. S14. As expected, the proportions of patients with indeterminate results increased compared to those observed with a cut-off for at least 95% of specificity. Nevertheless, it was always smaller for Agile 4 than FIB-4 and LSM in all datasets. The number of patients in the indeterminate zone for Agile 4 was moreover similar to that observed for LSM with a rule-in cut-off for at least 95% of specificity. Furthermore, even if the sensitivities decreased, the one of Agile 4 stayed superior to those of FIB-4 and LSM especially in the training set and the internal validation set and slightly in the NASH CRN and the French NAFLD cohorts. Finally, the PPV and the LR+ were highly increased especially in the internal validation set and the NASH CRN cohort (approximately 95% for Agile 4 vs 60% for FIB-4 and 80% for LSM) which would make it possible to give a quasi-certain diagnosis of cirrhosis.

Comparison of performances of FIB-4 [4] and LSM [5] using published cut-off values with Agile 3+ for the diagnosis of advanced fibrosis

According to [4], the rule-out cut-off for FIB-4 for the diagnosis of advanced fibrosis was 1.3 for patients < 65 years old and 2.0 for patients ≥ 65 years old and the rule-in cut-off was 2.67 for all patients. According to [5], the rule-out and rule-in cut-off values for LSM for the diagnosis of advanced fibrosis were 8.0 kPa and 12 kPa, respectively. The performances of FIB-4 and LSM with these cut-off values were computed and compared to Agile 3+ (Table S13). Although PPV in the rule-in zones of FIB-4 were higher than that achieved with LSM and Agile 3+ and with the results obtained with derived cut-off values (Table 2), their sensitivities were much lower and their numbers of patients with indeterminate results were higher. Moreover, concerning LSM, the number of patients with indeterminate results stayed higher and the sensitivity and the PPV in the rule-in zones remained lower than those of Agile 3+.

Comparison of AUROCs of Agile 4 and Agile 3+ for patients with BMI<30 kg/m² vs BMI≥30 kg/m²

AUROCs of Agile 4 and Agile 3+ for patients with BMI<30 kg/m² vs BMI≥30 kg/m² were compared using Delong test (at a two-sided 5% significance level) in order to evaluate if the scores performances were impacted by elevated BMI (Table S14). For Agile 4, no significant difference was observed between both groups evidencing that Agile 4 performs as well in obese patients and non-obese patients. For Agile 3+, although no significant difference was observed in the internal validation set, one was observed in the NASH CRN and the French NAFLD cohorts.

Comparison of AUROCs of Agile 4 and Agile 3+ for patients with steatosis grade S0/S1 vs S≥2

AUROCs of Agile 4 and Agile 3+ for patients with steatosis grade S0/S1 vs S≥2 were compared using Delong test (at a two-sided 5% significance level) in order to evaluate if the scores performances were impacted by steatosis. For both Agile 3+ and Agile 4, no significant difference was observed between both groups evidencing that Agile 3+ and Agile 4 perform as well in patients with steatosis grades S0/S1 and S≥2.

Comparison of AUROCs of Agile 4 and Agile 3+ for diabetic vs non-diabetic patients

AUROCs of Agile 4 and Agile 3+ for diabetic vs non-diabetic patients were compared using Delong test (at a two-sided 5% significance level) (Table S16). There was no significant difference of performances between both groups whether Agile 4 or Agile 3+ in the internal validation set and the NASH CRN cohort. Significant differences were observed for both scores in the French NAFLD cohort. However, while AUROCs in diabetic patients were lower than those of non-diabetic patients, they remained very good in this at risk population (AUROCs ≥ 0.80).

Comparison of AUROCs for Agile 4 and Agile 3+ for patients with LSM measured with M vs XL probe

AUROCs of Agile 4 and Agile 3+ for patients with LSM measured with M vs XL probe were compared using Delong test (at a two-sided 5% significance level) (Table S19). For Agile 4, no significant difference of performances between both groups was observed in the internal validation set, the NASH CRN and the French NAFLD cohorts. For Agile 3+, there was no significant difference of performance in the internal validation set only, while significant differences in the both external validation set. However, while AUROCs in patients with LSM measured with XL probe were lower than those of patients with LSM measured with M probe, they remained very good (AUROCs ≥ 0.80).

Performances of 90% sensitivity and 90% specificity cut-off values for the diagnosis of advanced fibrosis and cirrhosis

Rule-out cut-off values for at least 90% sensitivity and rule-in cut-off values for at least 90% specificity were derived on the training set for Agile 4, FIB-4 and LSM for the diagnosis of cirrhosis and Agile 3+, FIB-4 and LSM for the diagnosis of advanced fibrosis.

The cut-off values of Agile 4 were 0.169 and 0.388 for rule-out and rule-in, respectively, with characteristics detailed in Table S18. In comparison with results of Agile 4 in Table 2, less specificities of Agile 4 in the rule-out zone, less PPV and sensitivities in the rule-in zone were obtained. However, performances of Agile 4 remained better than those of FIB-4 and LSM.

The rule-out cut-off of Agile 3+ was 0.351 (rule-in cut-off being already achieved for at least 90% specificity In Table 2), with characteristics detailed in Table S18. In comparison with results of Agile 3+ in Table 2, less specificities of Agile 3+ in the rule-out zone and more patients with indeterminate results are obtained. However, performances of Agile 3+ remained better compared to FIB-4 and LS.

Comparison of AUROCs of Agile 4 and Agile 3+ for patients with LB with length > 15 mm vs length≤15 mm

AUROCs of Agile 4 and Agile 3+ for patients with LB with length > 15 mm vs length≤15 mm were compared using Delong test (at a two-sided 5% significance level) (Table S19). There was no difference of performances between both groups for Agile 4 and a very slight difference (very close to a 5%-significance level) between both groups for Agile 3+ in the internal validation set. No difference was observed for Agile 4 in the NASH CRN cohort but a significant difference was observed for Agile 3+. Moreover, no difference was observed between both whether Agile 4 or Agile 3+ in the French NAFLD cohort. Let's notice the imbalance between both subgroups as much in terms of number of patients as target prevalence that induce a bias for the comparisons.

Performances of sequential use of Agile scores after use of LSM alone compared to Agile score alone

The sequential use of LSM followed by Agile scores was performed as described in Fig. S19. The number of patients ruled-out, ruled-in and with indeterminate results using LSM followed by Agile or Agile scores alone are compared in Figs S20 and S21 on the training and validation sets; for advanced fibrosis and cirrhosis identification, respectively.

Supplementary figures

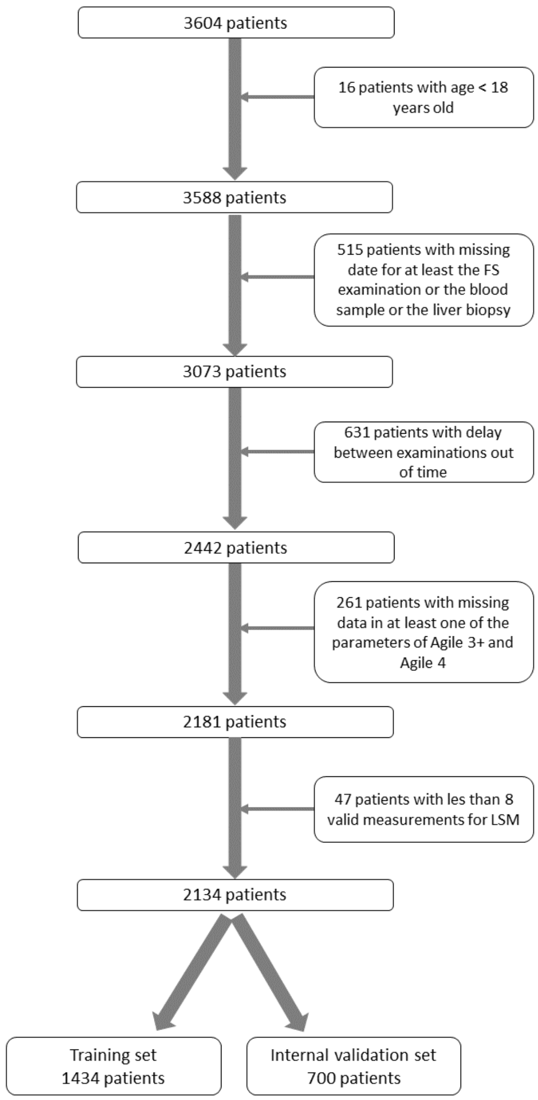

Fig. S1: Training and internal validation set flow chart

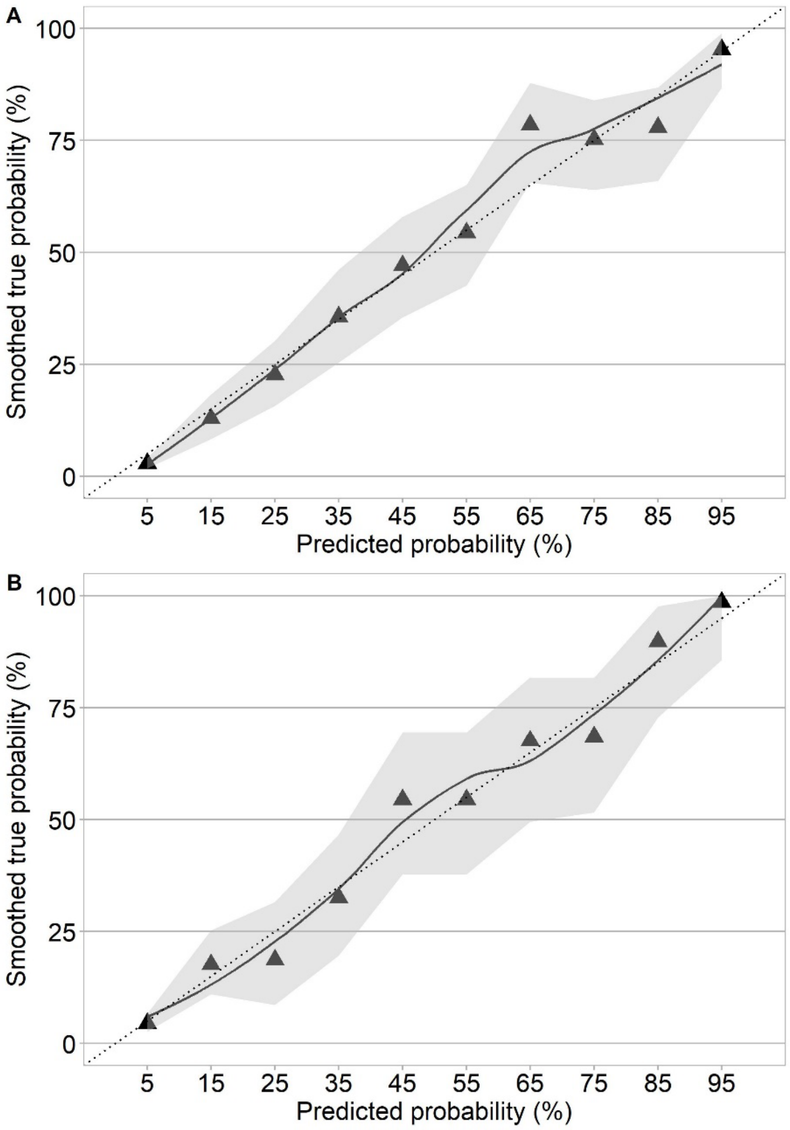

Fig. S2: Calibration plots for Agile 4 on the training set (A) and the internal validation set (B)

The calibration plot characterizes the agreement between observed proportion and predicted probabilities. Calibration of the data is estimated using a smoothed regression line using locally estimated scatterplot smoothing (LOESS) that allows inspection of the calibration across the range of predicted values and determination of whether there are segments of the range in which the model is poorly calibrated [6]. Triangles represent participants grouped by similar predicted risk. The dotted line represents the ideal calibration. The solid line is the calibration estimated on the data using locally estimated scatterplot smoothing (LOESS). The transparent grey area indicates 95% confidence interval.

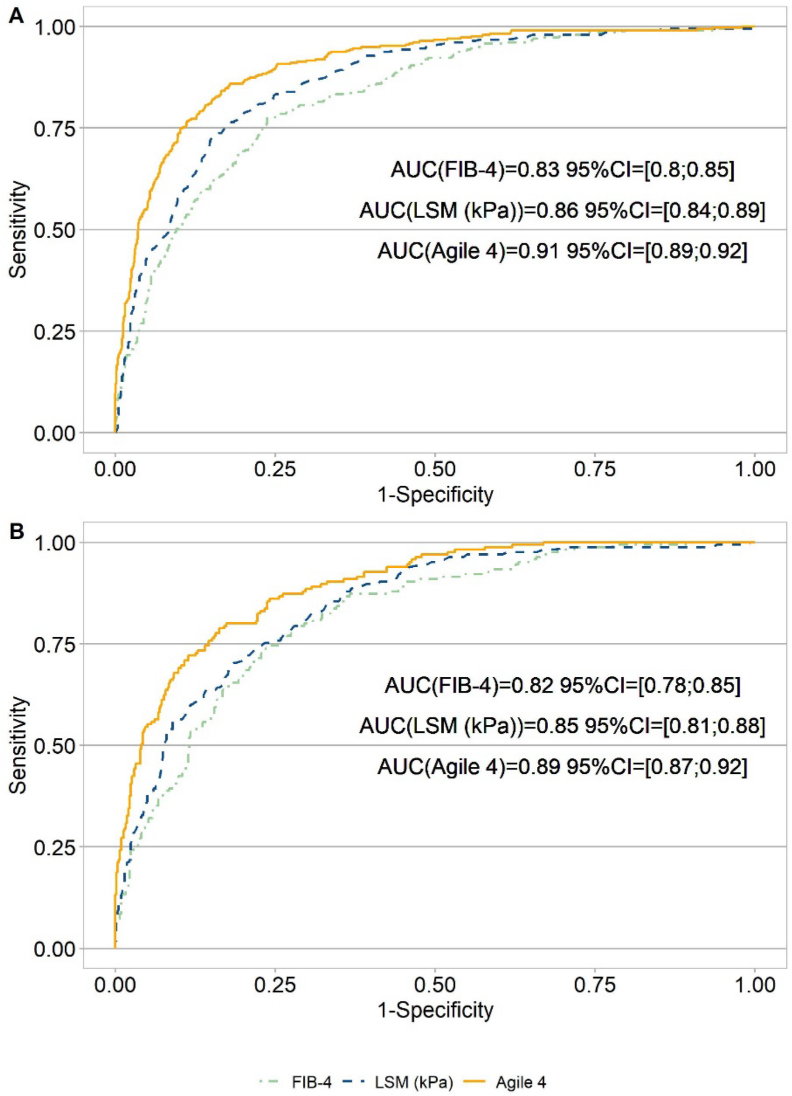

Fig. S3: ROC curves of FIB-4, liver stiffness measurement (LSM) and Agile 4 for the diagnosis of cirrhosis in the (A) training and (B) internal validation sets

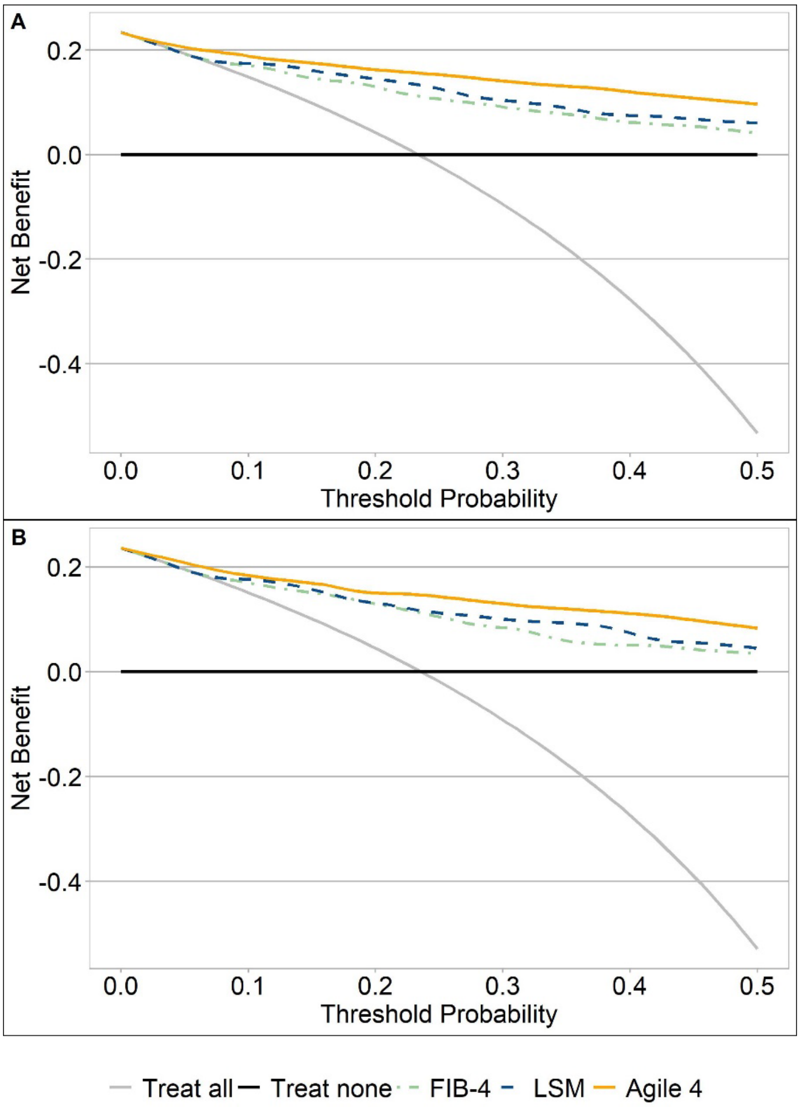

Fig. S4: Decision curves of FIB-4, liver stiffness measurement (LSM) and Agile 4 for the diagnosis of cirrhosis in comparison to default strategies of treating all patients as having ("Treat all") or not ("Treat none") cirrhosis in the (A) training and (B) internal validation sets

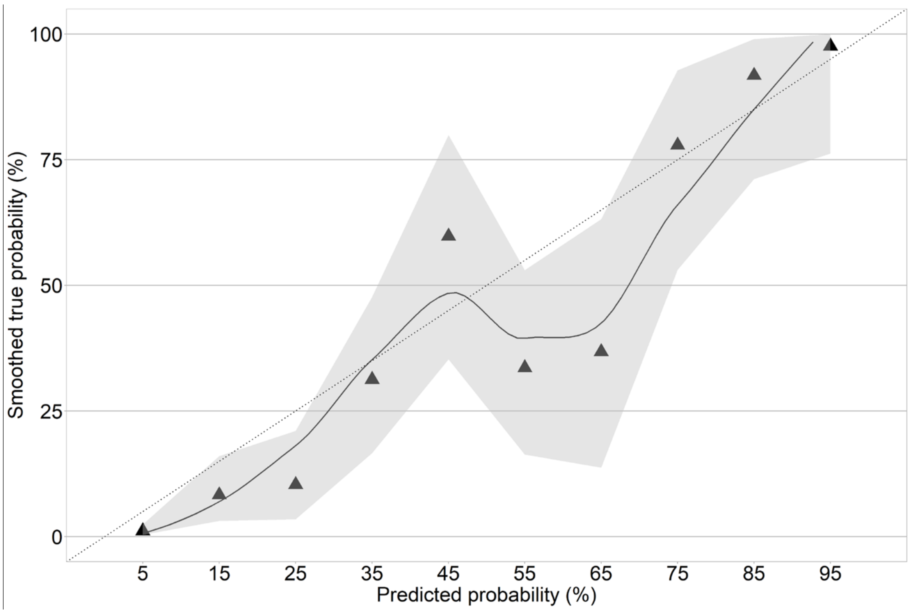

Fig. S5: Calibration plot for Agile 4 on the NASH CRN cohort

The calibration plot characterizes the agreement between observed proportion and predicted probabilities. Calibration of the data is estimated using a smoothed regression line using locally estimated scatterplot smoothing (LOESS) that allows inspection of the calibration across the range of predicted values and determination of whether there are segments of the range in which the model is poorly calibrated [6]. Triangles represent participants group by similar predicted risk. The dotted line represents the ideal calibration. The solid line is the calibration estimated on the data using locally estimated scatterplot smoothing (LOESS). The transparent grey area indicates 95% confidence interval.

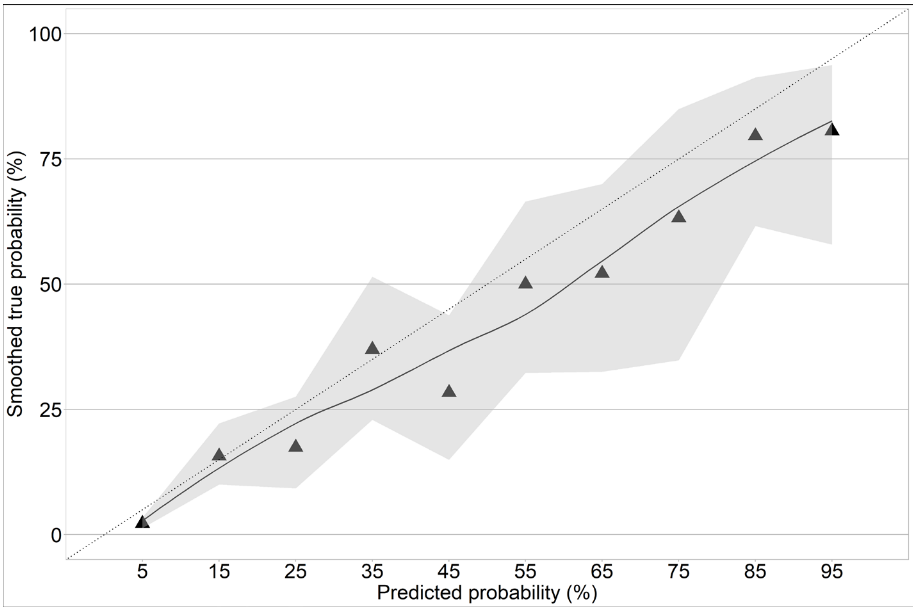

Fig. S6: Calibration plot for Agile 4 on the French NAFLD cohort

The calibration plot characterizes the agreement between observed proportion and predicted probabilities. Calibration of the data is estimated using a smoothed regression line using locally estimated scatterplot smoothing (LOESS) that allows inspection of the calibration across the range of predicted values and determination of whether there are segments of the range in which the model is poorly calibrated [6]. Triangles represent participants group by similar predicted risk. The dotted line represents the ideal calibration. The solid line is the calibration estimated on the data using locally estimated scatterplot smoothing (LOESS). The transparent grey area indicates 95% confidence interval.

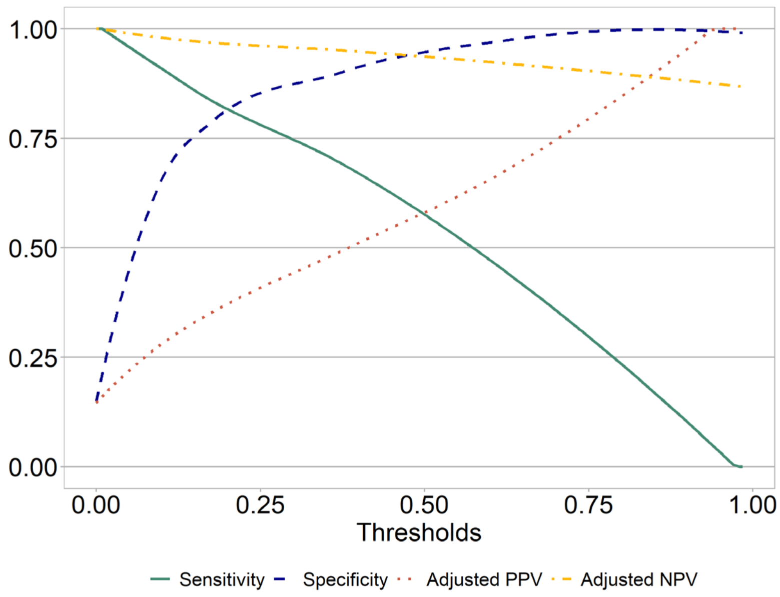

Fig. S7: Sensitivity, specificity, adjusted positive predictive value (PPV) and adjusted negative predictive value (NPV) of Agile 4 for the diagnosis of cirrhosis in the internal validation set

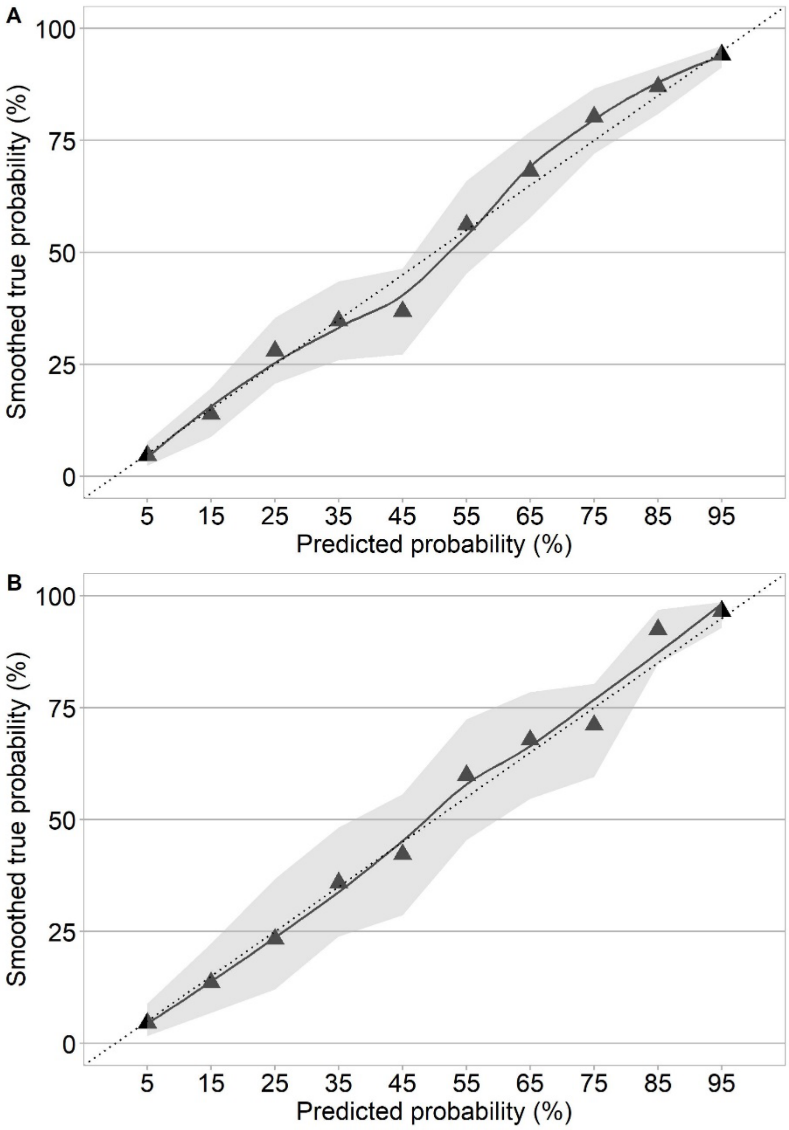

Fig. S8: Calibration plots for Agile 3+ on the training (A) and the internal validation sets (B)

The calibration plot characterizes the agreement between observed proportion and predicted probabilities. Calibration of the data is estimated using a smoothed regression line using locally estimated scatterplot smoothing (LOESS) that allows inspection of the calibration across the range of predicted values and determination of whether there are segments of the range in which the model is poorly calibrated [6]. Triangles represent participants group by similar predicted risk. The dotted line represents the ideal calibration. The solid line is the calibration estimated on the data using locally estimated scatterplot smoothing (LOESS). The transparent grey area indicates 95% confidence interval.

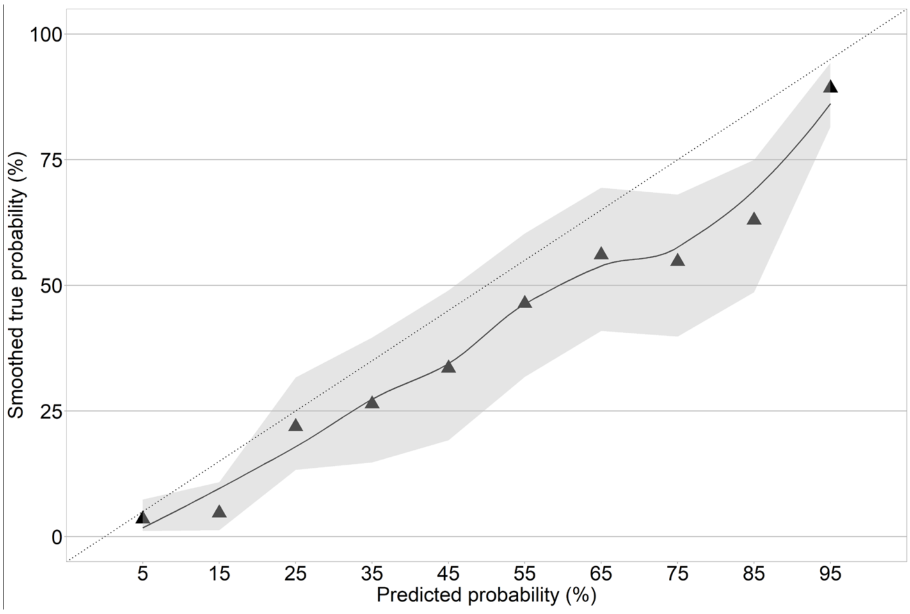

Fig. S9: Calibration plot for Agile 3+ on the NASH CRN cohort

The calibration plot characterizes the agreement between observed proportion and predicted probabilities. Calibration of the data is estimated using a smoothed regression line using locally estimated scatterplot smoothing (LOESS) that allows inspection of the calibration across the range of predicted values and determination of whether there are segments of the range in which the model is poorly calibrated [6]. Triangles represent participants group by similar predicted risk. The dotted line represents the ideal calibration. The solid line is the calibration estimated on the data using locally estimated scatterplot smoothing (LOESS). The transparent grey area indicates 95% confidence interval.

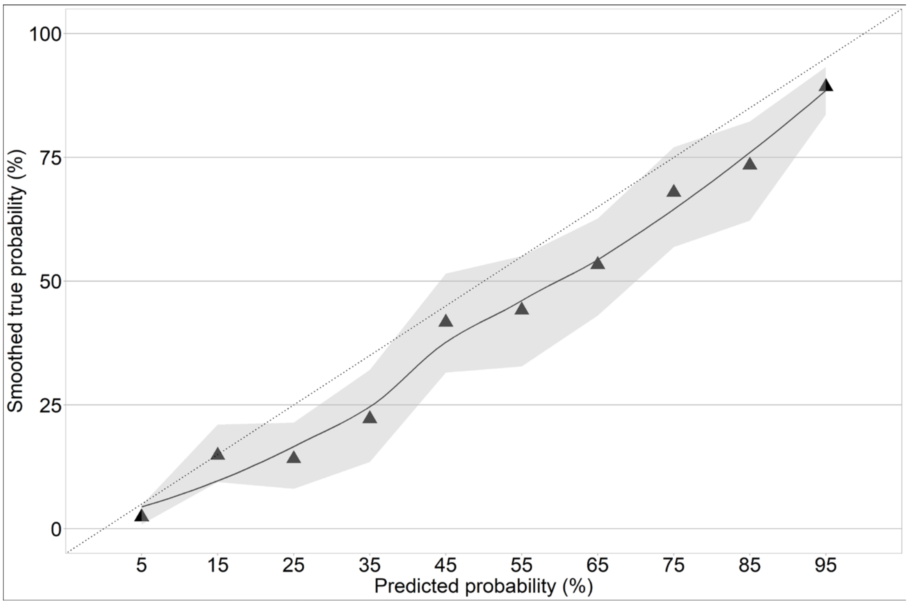

Fig. S10: Calibration plot for Agile 3+ on the French NAFLD cohort

The calibration plot characterizes the agreement between observed proportion and predicted probabilities. Calibration of the data is estimated using a smoothed regression line using locally estimated scatterplot smoothing (LOESS) that allows inspection of the calibration across the range of predicted values and determination of whether there are segments of the range in which the model is poorly calibrated [6]. Triangles represent participants group by similar predicted risk. The dotted line represents the ideal calibration. The solid line is the calibration estimated on the data using locally estimated scatterplot smoothing (LOESS). The transparent grey area indicates 95% confidence interval.

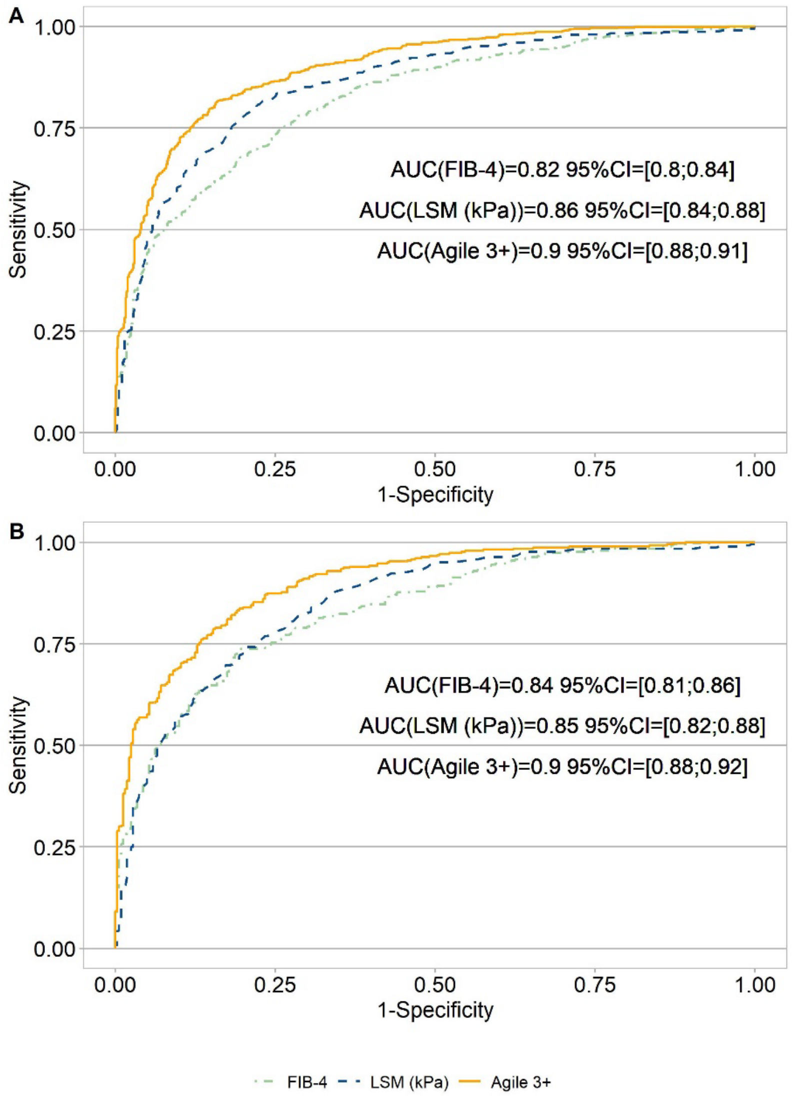

Fig. S11: ROC curves of FIB-4, liver stiffness measurement (LSM) and Agile 3+ for the diagnosis of advanced fibrosis in (A) training and (B) internal validation sets

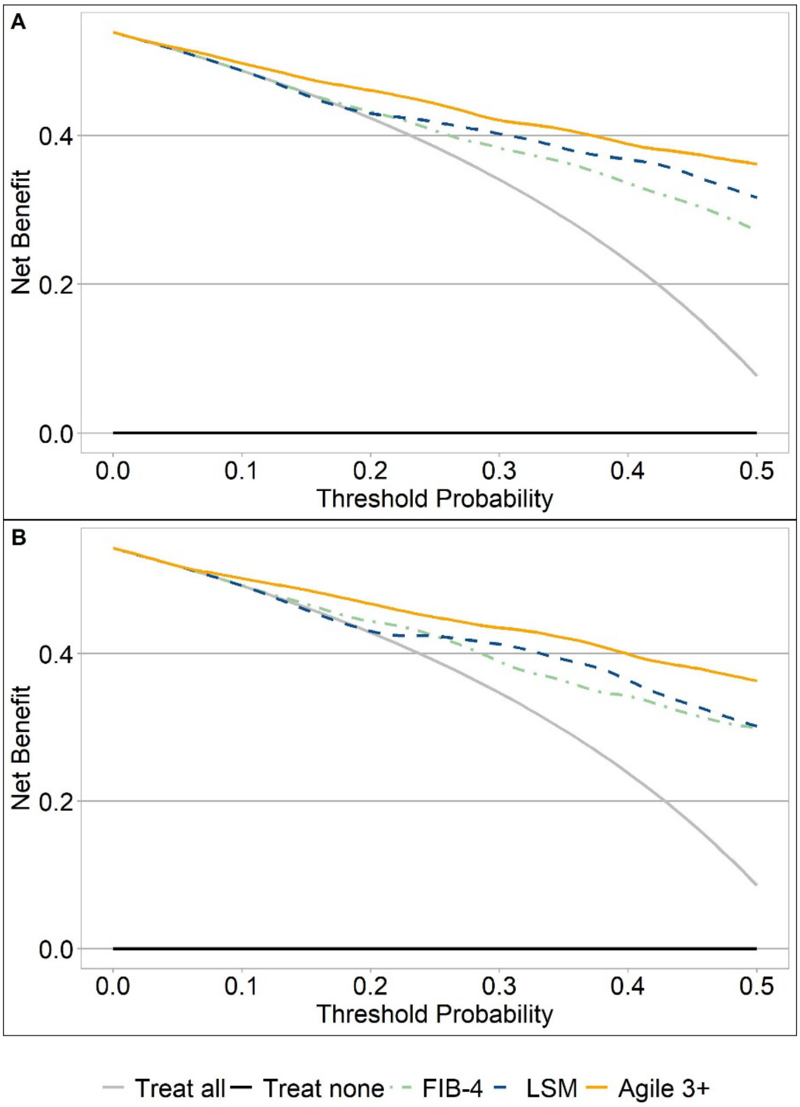

Fig. S12: Decision curves of FIB-4, liver stiffness measurement (LSM) and Agile 3+ for the diagnosis of advanced fibrosis in comparison to default strategies of treating all patients as having ("Treat all") or not ("Treat none") AF in the (A) training and (B) internal validation sets

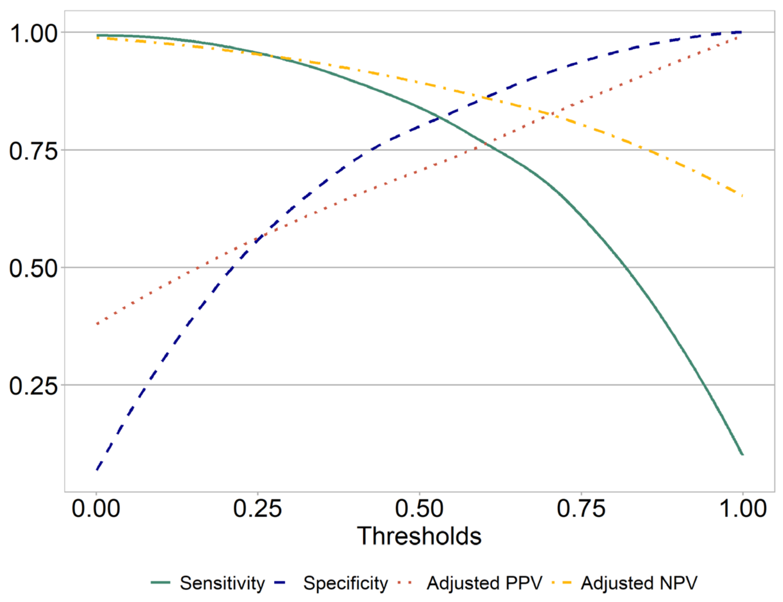

Fig. S13: Sensitivity, specificity, adjusted positive predictive value (PPV) and adjusted negative predictive value (NPV) of Agile 3+ for the diagnosis of advanced fibrosis in the internal validation set

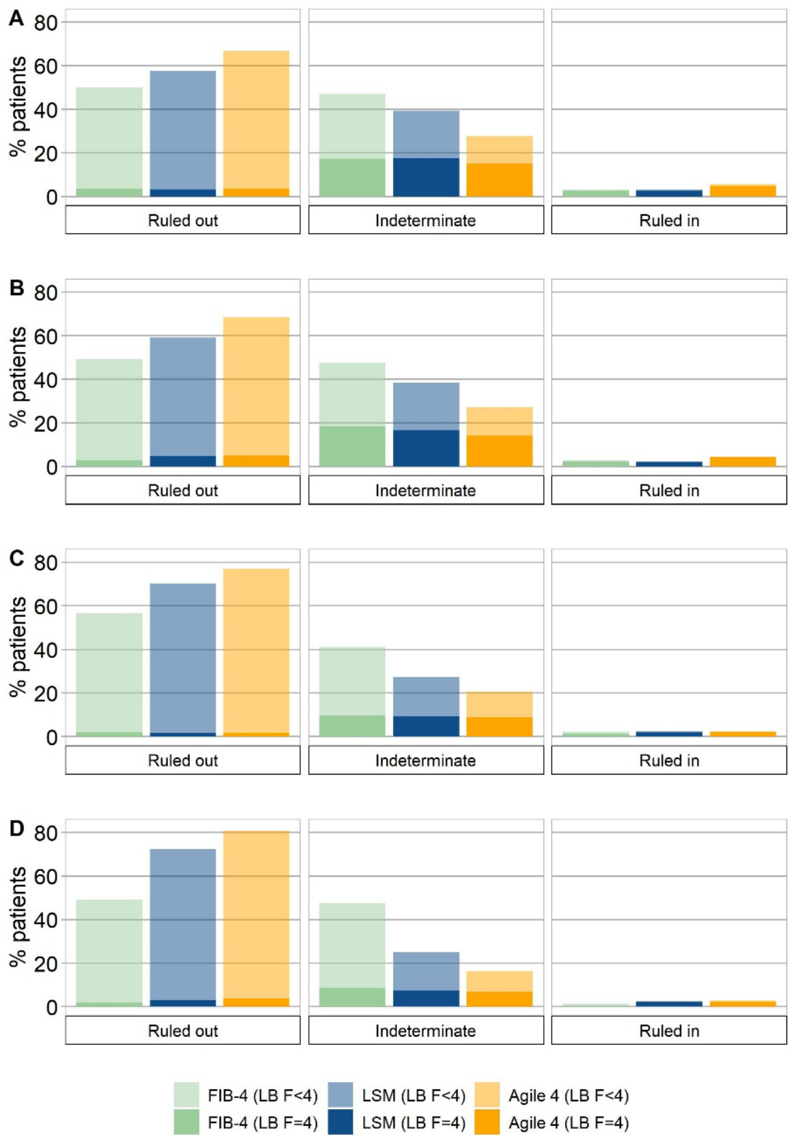

Fig. S14: Percentage of patients in rule-out (<85% Sensitivity cut-off), indeterminate and rule-in zones (≥99% Specificity cut-off) for the diagnosis of cirrhosis with FIB-4, LSM and Agile 4

(A) Training set (n=1434), (B) Internal validation set (n=700), (C) NASH CRN cohort (n=585), (D) French NAFLD cohort (n=1042). Results of FIB-4, LSM and Agile 4 are represented in green, blue and yellow, respectively. On each bar, the solid and transparent parts represent the percentage of patients with and without cirrhosis according to liver biopsy (LB), respectively.

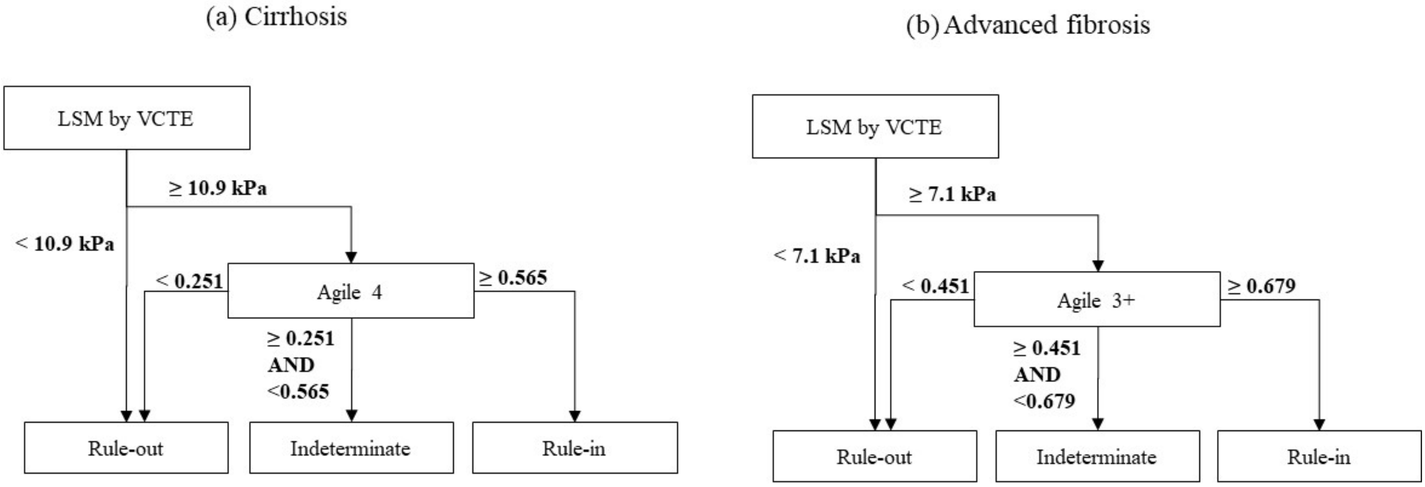

Fig. S15: Sequential use of LSM by VCTE followed by Agile scores for the identification of patients with (a) cirrhosis and (b) advanced fibrosis.

Ruled out cut-off values for LSM by VCTE are with 90% Sensivity according to Eddowes et al. (4).

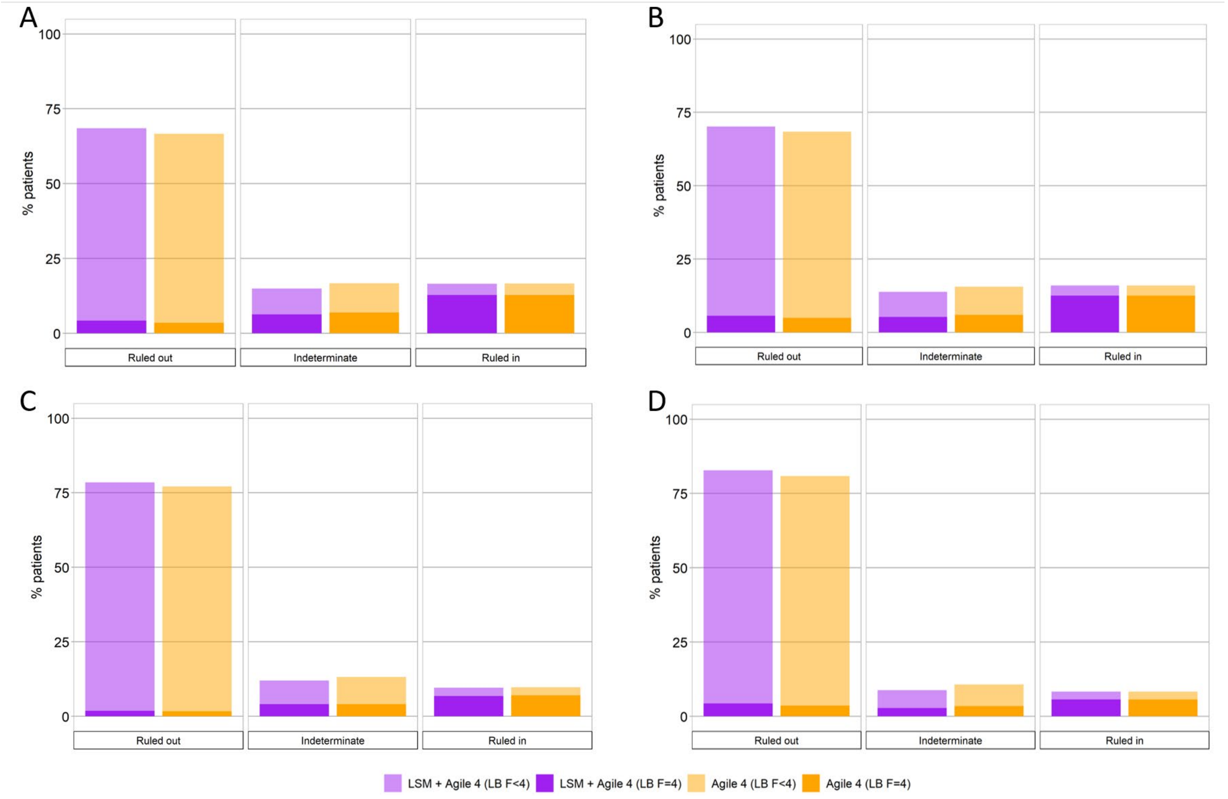

Fig. S16: Percentage of patients in rule-out, indeterminate and rule-in-zone with a sequential use of LSM followed by Agile 4 versus Agile 4 alone for the diagnosis of cirrhosis.

(A) Training set (n=1434), (B) Internal validation set (n=700), (C) NASH CRN cohort (n=585), (D) French NAFLD cohort (n=1042). Results of LSM + Agile 4 and Agile 4 are represented in purple and yellow, respectively. On each bar, the solid and transparent parts represent the percentage of patients with and without advanced fibrosis according to liver biopsy (LB), respectively.

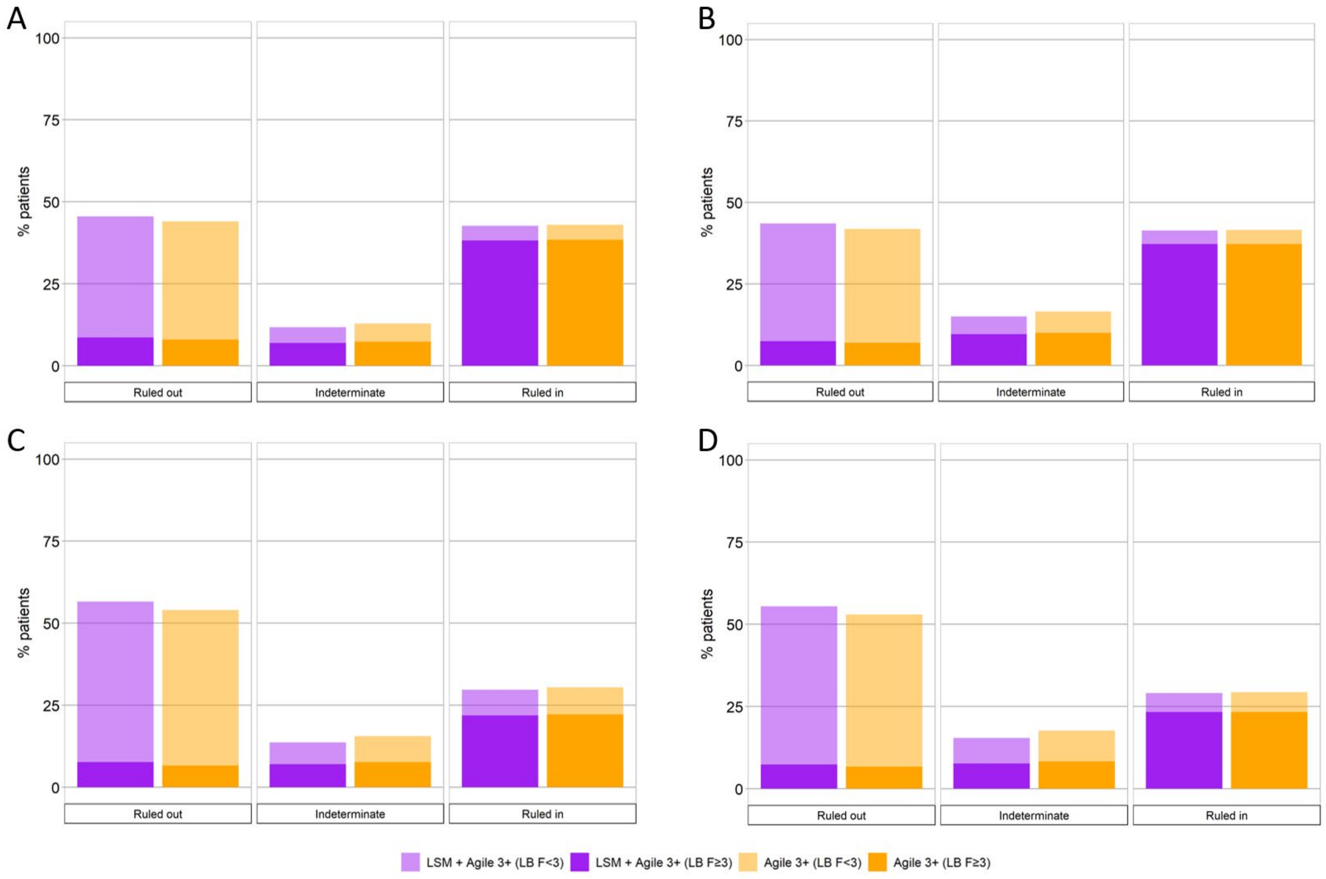

Fig. S17: Percentage of patients in rule-out, indeterminate and rule-in-zone with a sequential use of LSM followed by Agile 3+ versus Agile 3+ alone for the diagnosis of advanced fibrosis.

(A) Training set (n=1434), (B) Internal validation set (n=700), (C) NASH CRN cohort (n=585), (D) French NAFLD cohort (n=1042). Results of LSM + Agile 3+ and Agile 3+ are represented in purple and yellow, respectively. On each bar, the solid and transparent parts represent the percentage of patients with and without advanced fibrosis according to liver biopsy (LB), respectively.

Supplementary tables

Table S1: Transparent reporting of a multivariable prediction model for individual prognosis or diagnosis TRIPOD checklist

Section/Topic	Item	Checklist Item	Page
**Title and abstract**			
Title	1	Identify the study as developing and/or validating a multivariable prediction model, the target population, and the outcome to be predicted.	1
Abstract	2	Provide a summary of objectives, study design, setting, participants, sample size, predictors, outcome, statistical analysis, results, and conclusions.	3
**Introduction**			
Background and objectives	3a	Explain the medical context (including whether diagnostic or prognostic) and rationale for developing or validating the multivariable prediction model, including references to existing models.	4
	3b	Specify the objectives, including whether the study describes the development or validation of the model or both.	4
**Methods**			
Source of data	4a	Describe the study design or source of data (e.g., randomized trial, cohort, or registry data), separately for the development and validation data sets, if applicable.	5<br>Table S2<br>Table S3
	4b	Specify the key study dates, including start of accrual; end of accrual; and, if applicable, end of follow-up.	Table S2<br>Table S3
Participants	5a	Specify key elements of the study setting (e.g., primary care, secondary care, general population) including number and location of centres.	5<br>Table S2<br>Table S3
	5b	Describe eligibility criteria for participants.	6<br>Table S2<br>Table S3
	5c	Give details of treatments received, if relevant.	Not applicable
Outcome	6a	Clearly define the outcome that is predicted by the prediction model, including how and when assessed.	7
	6b	Report any actions to blind assessment of the outcome to be predicted.	7<br>Table S2<br>Table S3
Predictors	7a	Clearly define all predictors used in developing or validating the multivariable prediction model, including how and when they were measured.	8-9<br>Table 1<br>Table S2<br>Table S3
	7b	Report any actions to blind assessment of predictors for the outcome and other predictors.	5
Sample size	8	Explain how the study size was arrived at.	7-8
Missing data	9	Describe how missing data were handled (e.g., complete-case analysis, single imputation, multiple imputation) with details of any imputation method.	Cases with missing data were excluded.
	10a	Describe how predictors were handled in the analyses.	7-8

Statistical analysis methods	10b	Specify type of model, all model-building procedures (including any predictor selection), and method for internal validation.	7-8
	10c	For validation, describe how the predictions were calculated.	7-8
	10d	Specify all measures used to assess model performance and, if relevant, to compare multiple models.	7-8
	10e	Describe any model updating (e.g., recalibration) arising from the validation, if done.	Not applicable
Risk groups	11	Provide details on how risk groups were created, if done.	Not applicable
Development vs. validation	12	For validation, identify any differences from the development data in setting, eligibility criteria, outcome, and predictors.	8-9
**Results**			
Participants	13a	Describe the flow of participants through the study, including the number of participants with and without the outcome and, if applicable, a summary of the follow-up time. A diagram may be helpful.	Table S2<br>Table S3
	13b	Describe the characteristics of the participants (basic demographics, clinical features, available predictors), including the number of participants with missing data for predictors and outcome.	Table 1
	13c	For validation, show a comparison with the development data of the distribution of important variables (demographics, predictors and outcome).	Table 1
Model development	14a	Specify the number of participants and outcome events in each analysis.	Table 1
	14b	If done, report the unadjusted association between each candidate predictor and outcome.	Not applicable
Model specification	15a	Present the full prediction model to allow predictions for individuals (i.e., all regression coefficients, and model intercept or baseline survival at a given time point).	9, 11
	15b	Explain how to the use the prediction model.	9, 11
Model performance	16	Report performance measures (with CIs) for the prediction model.	9-12<br>Table 2<br>Table 3
Model-updating	17	If done, report the results from any model updating (i.e., model specification, model performance).	Not applicable
**Discussion**			
Limitations	18	Discuss any limitations of the study (such as nonrepresentative sample, few events per predictor, missing data).	13-14
Interpretation	19a	For validation, discuss the results with reference to performance in the development data, and any other validation data.	12-14
	19b	Give an overall interpretation of the results, considering objectives, limitations, results from similar studies, and other relevant evidence.	12-14
Implications	20	Discuss the potential clinical use of the model and implications for future research.	12-14
**Other information**			

Supplementary information	21	Provide information about the availability of supplementary resources, such as study protocol, Web calculator, and data sets.	Not applicable
Funding	22	Give the source of funding and the role of the funders for the present study.	3, 8

Table S2: Description of the cohorts included in the internal dataset (training and internal validation sets).

		China Hong-Kong NAFLD cohort	China Wenzhou NAFLD cohort	Malaysian NAFLD cohort	Turkish NAFLD cohort	UK NAFLD cohort	Canadian overweight cohort	Clinical trial cohort
**Study description**	**Funding**	Grant from the Research grant Council of Hong-Kong government	Training funding by the High level creative Talents from Department of public health in Zhejiang province	Research grant from the university of Malaya	Scientific research fund from Marmara university	Echosens and UK Nationale Institute for Health Research (NIHR)	Echosens	Gilead Sciences
	**Enrolment dates (first and last inclusion)**	From 2003/05 to 2017/11	From 2017/01 to 2018/03	From 2012/11 to 2015/10 and from 2016/09 to 2018/03	From 2016/01 to 2018/09	From 2014/03 to 2017/01	From 2009/07 to 2010/07	Jan 2017 to Mar 2018
	**Study design**	Prospective cross-sectional single centre study	Prospective cross-sectional single centre study·	Prospective cross-sectional single centre study	Prospective cross-sectional single centre study	Prospective cross-sectional multicenter study	Prospective multicenter study	Prospective, multicenter, international, double-blind, randomized, placebo-controlled clinical trials (use of baseline and screen failure data only)
	**PMID if data were used for publication**	PMID-30658987	NA	PMID-24548002	NA	PMID-32027858	PMID-22435761	PMID-32147362

		PMID-28506907 PMID-23032979 PMID-2010754 PMID-30658997		PMID-25788185 PMID-25184298 PMID-31442603 PMID-31310032		PMID-30689971	PMID-22027584 PMID-21898479	PMID-31271665
	**Center description**	Hepatology tertiary care	Hepatology tertiary care	Hepatology tertiary care	Hepatology tertiary care	Hepatology tertiary care	Hepatology tertiary care	Outpatient clinics including tertiary and secondary care centers.
	**Eligibility criteria**	Inclusion: age > 18 years; biopsy-proven NAFLD· Exclusion: other liver disease; excessive alcohol consumption (>20g/day in women and > 30g/day in men); secondary fatty liver (e·g· DILI); history of liver surgery or liver transplantation; history of HCC;	Inclusion: age 18-75 years; BMI < 35 kg/m^2; US, CT or MRI imaging showing fatty liver disease; abnormal ALT but below 5 ULN; no alcohol drinking history or daily alcohol intake < 20 g for male and 10 g for female. Exclusion: decompensated cirrhosis; history of HCC,	Inclusion: NAFLD patients diagnosed on US following exclusion of other cause of CLD including alcohol (>14 units per week in women and >21 units per week in men). Exclusion: decompensated cirrhosis; history of HCC, patients on liver transplant list	Inclusion: evidence of hepatic steatosis on US; abnormal liver enzymes or organomegaly; absence of secondary causes of hepatic fat accumulation (e.g. significant alcohol consumption (>20g/day in women and >30g/day in men) and previous use of	Inclusion: age > 18 years; biopsy-proven NAFLD within 2 weeks before or after FibroScan examination; negative for hepatitis B surface antigen, anti-hepatitis C virus antibody, hepatitis C virus RNA, and hepatitis B virus DNA. Exclusion: ascites, pregnancy, active	Inclusion: age > 18 years; BMI ≥ 28kg/m^2; liver biopsy within 1 month after the enrollment or 6 months before the enrollment; abdominal ultrasound technically possible. Exclusion: Confirmed diagnosis and/or history of malignancy, or other terminal disease; ascites;	Clinical suspicion of NASH with F3-F4 fibrosis; no prior history of hepatic decompensation , transplantation, or HCC; MELD <=12; Child-Pugh A; ALT <10 x ULN; HbA1c <9%, no excessive alcohol consumption (>14 units/week in women and >21 units/week in men)

		history of malignancy unless if complete remission > 5 years; decompensated cirrhosis; patients on liver transplant list	patients on liver transplant list		steatogenic drugs)· Exclusion: patients with viral hepatitis, DILI, autoimmune hepatitis, metabolic/genetic liver disease or low platelets count (< 100 x 10^9/L), history of malignancy and heart failure; decompensated cirrhosis; history of HCC, patients on liver transplant list	implantable medical device (such as pacemaker or defibrillator), liver transplantation, cardiac failure or clinically significant valvular disease, haemochromatosis, refusal to have liver biopsy or blood tests, alcohol consumption above recommended limits (> 14 units per week for women and > 21 units per week for men), diagnosis of active malignancy or other terminal disease, or participation in another clinical trial within the previous 30 days.	pregnancy; active implantable medical device (such as pacemaker or defibrillator); liver transplantation; patient with heart disease; refusal to undergo a liver biopsy; excessive alcohol consumption (>10g/day in women and > 20g/day in men)	

**FibroScan device information**	**Probe used**	M and XL	M only	M only	M and XL	M and XL	M and XL	M and XL
	**Probe selection**	Both probes on all patients	NA	NA	Automatic probe selection tool	Automatic probe selection tool	Both probes on all patients	M or XL, depending on patient characteristics. In some cases, devices with automatic probe selection tool used.
	**Number of FibroScan operators and experience**	N=4 Experience >100 VCTE examinations for all	N=1 5 years' experience	N=2 Experience >200 VCTE examinations for both	N=1 Experience >10000 VCTE examinations	N = 14	N = 10	At least one experienced operator per site. No specific level of experience explicitly stated in protocol.
	**Patients fasting for at least 3 hours?**	Yes	Yes, more than 95%	Yes	Yes	Yes	No (not recommended at the time the study was conducted)	This was the recommendation but the information was not recorded.
	**Median time interval in days (IQR) between FS examination and LB**	1(16.8)	0(2)	0(0)	6(23)	0 (7)	34 (37)	37 (36)
**Histological information**	**Reason to send a patient to LB**	Persistent elevated transaminase, high metabolic burden	Persistent elevated transaminase or elevated LSM by VCTE or	Persistent ALT or AST ≥ 40 or reasons for NASH to be suspected (e·g·	Evidence of hepatic steatosis on US, abnormal liver enzymes or	Abnormal liver enzymes and an ultrasound scan showing an echobright liver	Standard of care	Clinical suspicion of NASH with F3-F4 fibrosis

		suspicious of advanced disease, elevated LSM by VCTE	CAP (especially LSM)	significant fibrosis at LSM by VCTE, obese patient with metabolic syndrome)	organomegaly, absence of secondary causes of hepatic fat accumulation (e·g· significant alcohol consumption and previous use of steatogenic drugs), LSM by VCTE > 6 kPa or rarely patients with LSM by VCTE < 6 kPa to exclude other CLD			
	**LB reading**	Central reading by a single expert pathologist	Routine reading by a single expert pathologist	Central reading by a single expert pathologist	Central reading by a single expert pathologist	Independent reading by two experienced pathologists. In case of disagreement, they reviewed together to reach consensus.	Central reading by two expert pathologists. In case of disagreement, they reviewed together to reach consensus	Central reading by a single expert pathologist
	**Type of needle**	16G	16G	18G	16G	Clinical routine	Clinical routine	No. It was advised but it was up to the sites. The

								information was not collected.

Table S3: Description of the external validation cohorts

		NASH CRN cohort	French NAFLD cohort
**Study description**	**Funding**	NIH NIDDK	Angers University Hospital<br>Bordeaux University Hospital<br>Grenobles Alpes University Hospital
	**Enrolment dates (first and last inclusion)**	11 July 2014 –<br>27 March 2019	2004-2019
	**Study design**	Prospective cohort study	Prospective cross sectional multicenter study
	**PMID if data were used for publication**	PMID-29705261<br>PMID-28859228	PMID-33307138<br>PMID-33236409<br>PMID-26659452<br>PMID-31102719<br>PMID-27151181
	**Center description**	Tertiary care centers	Hepatology tertiary care
	**Eligibility criteria**	Inclusion: At least 18 years of age; written informed consent; Liver biopsy ≤120 days prior to enrollment (or previously enrolled in prior NASH CRN study); collection of serum and plasma within 90 days of enrollment and liver biopsy; absence of regular or excessive use (AUDIT≥7) of alcohol in prior 2 years; willingness to be in study for ≥1 year.<br>Exclusion: Clinical/histologic evidence of alcoholic liver disease; other chronic liver disease; history of prolonged parenteral nutrition; short bowel syndrome; history of biliopancreatic diversion; history of bariatric surgery; known HIV positive; other condition likely to interfere with study follow-up; decompensated cirrhosis;	Inclusion: Patients with biopsy-proven NAFLD and no other concomitant cause of chronic liver disease<br>Exclusion: decompensated cirrhosis; history of HCC, patients on liver transplant list; excessive alcohol consumption (> 210 g/week for men and 140 g /week for women)

		history of HCC, patients on liver transplant list Exclusion Criteria specific to FibroScan: Use of implantable active medical device;wound near application site of FibroScan pregnancy ascites	
**FibroScan device informatio n**	**Probe used**	M and XL	M and XL
	**Probe selection**	Automatic probe selection tool	M probe if BMI <30, XL probe if BMI >30
	**Number of FibroScan operators and experience**	Operators were trained and certified by Echosens North America. To be certified, 40-60 scans on 4-6 patients were required. There were 50 certified FibroScan operators.	In each investigating center: experienced nurses/MD with more than >500 FibroScan examination each one
	**Patients fasting for at least 3 hours?**	Yes	Angers & Bordeaux: Yes<br>Grenoble: outpatients, fasting not controlled
	**Median time interval in days (IQR) between FS examination and LB**	43 (54)	0 (0)
**Histological informatio n**	**Reason to send a patient to LB**	1. To evaluate suspected NASH<br>2. To evaluate disease status in patients known to have NAFLD	Abnormal liver function tests, hyperferritinaemia, metabolic syndrome, abnormal non-invasive tests of liver fibrosis (FIB-4, NFS, FibroMeter, LSM by VCTE)
	**LB reading**	Histology data were based on consensus readings by 9-member NASH CRN Pathology Committee, who were blinded to LSM and other biologicial/clinical data, study, and visit.	In each center, liver biopsy was read by an experienced pathologist expert in chronic liver disease
	**Type of needle**	15 to 18G	Angers & Bordeaux: 16G<br>Grenoble: 17G

Table S4: Patients' characteristics of training set

	F<4		F=4		F<3		F≥3	
	**Median (IQR) or n (%)**	**N**	**Median (IQR) or n (%)**	**N**	**Median (IQR) or n (%)**	**N**	**Median (IQR) or n (%)**	**N**
**Demographics**								
Age (years)	53.0 (17.0)	1099	59.0 (11.0)	335	50.0 (17.8)	662	59.0 (12.0)	772
Male sex	594 (54.0)	1099	135 (40.3)	335	387 (58.5)	662	342 (44.3)	772
BMI (kg/m²)	31.3 (7.70)	1017	32.8 (9.02)	308	30.5 (7.10)	590	32.6 (8.20)	735
**Metabolic**								
Diabetes (type 1 and 2)	506 (46.0)	1099	217 (64.8)	335	215 (32.5)	662	508 (65.8)	772
Hypertension	520 (47.3)	1099	199 (59.4)	335	245 (37)	662	474 (61.4)	772
**Blood**								
AST (U/L)	38.0 (30.5)	1099	44.0 (29.0)	335	34.0 (25.8)	662	44.0 (34.0)	772
ALT (U/L)	51 .0(51.5)	1099	44.0 (31.0)	335	49.0 (52.0)	662	49.0 (42.0)	772
AAR	0.750 (0.342)	1099	1.00 (0.401)	335	0.704 (0.318)	662	0.903 (0.417)	772
Platelets count (G/L)	234 (87.5)	1099	156 (74.0)	335	244 (84.0)	662	191 (91.2)	772
HDL (mmol/L)	1.11 (0.379)	835	1.24 (0.491)	279	1.11 (0.37)	462	1.16 (0.44)	652
LDL (mmol/L)	2.66 (1.33)	815	2.33 (1.11)	273	2.90 (1.29)	457	2.41 (1.22)	631
Albumin (g/L)	45.0 (4.00)	1008	44.0 (4.00)	330	45.0 (5.00)	603	44.0 (5.00)	735
GGT (UI/L)	54.0 (61.5)	1007	78.0 (94.8)	330	49.0 (56.8)	602	66.0 (80.0)	735
Triglycerides (mmol/L)	1.72 (1.08)	840	1.52 (0.83)	279	1.70 (1.02)	464	1.63 (1.04)	655
Fasting glucose (mmol/L)	6.00 (2.11)	992	6.55 (2.91)	323	5.69 (1.93)	594	6.49 (2.61)	721
**Non-invasive tests**								
FIB-4	1.20 (0.851)	1099	2.56 (1.82)	335	0.992 (0.648)	662	1.93 (1.52)	772
LSM by VCTE (kPa)	9.10 (6.65)	1099	21.1 (16.0)	335	7.40 (3.88)	662	15.7 (11.8)	772
**Fibrosis stage**								
NASH CRN scoring		1099		335		662		772
system	202 (18.4)		-		202 (30.5)		-	
F0	269 (24.5)		-		269 (40.6)		-	
F1	191 (17.4)		-		191 (28.9)		-	
F2	437 (39.8)		-		-		437 (56.6)	
F3	-		335 (100)		-		335 (43.4)	
F4								

Table S5: Patients' characteristics of internal validation set

	F<4		F=4		F<3		F≥3	
	**Median (IQR) or n (%)**	**N**	**Median (IQR) or n (%)**	**N**	**Median (IQR) or n (%)**	**N**	**Median (IQR) or n (%)**	**N**
**Demographics**								
Age (years)	54.0 (17.0)	535	60.0 (13.0)	165	52.0 (18.2)	320	59.0 (14.0)	380
Male sex	287 (53.6)	535	72.0 (43.6)	165	179 (55.9)	320	180 (52.6)	380
BMI (kg/m²)	31.3 (7.90)	495	32.4 (8.10)	151	31.0 (8.00)	289	32.0 (8.40)	357
**Metabolic**								
Diabetes (type 1 and 2)	252 (47.1)	535	105 (63.6)	165	112 (35.0)	320	245 (64.5)	380
Hypertension	247 (46.2)	535	97 (58.8)	165	114 (35.6)	320	230 (60.5)	380
**Blood**								
AST (U/L)	37.0 (27.0)	535	44.0 (29.0)	165	33.5 (24.2)	320	43.0 (30.2)	380
ALT (U/L)	48.0 (48.0)	535	42.0 (33.0)	165	48.5 (47.5)	320	45.0 (41.5)	380
AAR	0.781 (0.364)	535	1.02 (0.500)	165	0.701 (0.333)	320	0.926 (0.406)	380
Platelets count (G/L)	235 (85.5)	535	160 (83.0)	165	251 (84.2)	320	192 (90.8)	380
HDL (mmol/L)	1.11 (0.38)	401	1.15 (0.427)	140	1.09 (0.347)	218	1.14 (0.414)	323
LDL (mmol/L)	2.64 (1.29)	394	2.38 (1.20)	136	2.74 (1.30)	217	2.40 (1.15)	313
Albumin (g/L)	45.0 (5.00)	491	44.0 (5.00)	163	45.0 (5.00)	292	44.0 (4.00)	362
GGT (UI/L)	55.0 (60.5)	491	86.0 (102)	163	51.5 (55.2)	292	70.0 (78.8)	362
Triglycerides (mmol/L)	1.70 (1.04)	405	1.53 (0.92)	140	1.61 (0.96)	221	1.66 (1.04)	324
Fasting glucose (mmol/L)	5.9 (2.38)	484	6.55 (2.39)	161	5.7 (1.78)	286	6.61 (2.89)	359
**Non-invasive tests**								
FIB-4	1.18 (0.961)	535	2.41 (1.79)	165	0.988 (0.676)	320	1.94 (1.49)	380
LSM by VCTE (kPa)	8.90 (6.80)	535	20.9 (15.2)	165	7.00 (4.60)	320	14.4 (11.1)	380
**Fibrosis stage**								
NASH CRN scoring		535		165		320		380
system	97 (18.1)		-		97 (30.3)		-	
F0	130 (24.3)		-		130 (40.6)		-	
F1	93 (17.4)		-		93 (29.1)		-	
F2	215 (40.2)		-		-		215 (56.6)	
F3	-		165 (100)		-		165 (43.4)	
F4								

Table S6: Patients' characteristics of NASH CRN cohort

	F<4		F=4		F<3		F≥3	
	**Median (IQR) or n (%)**	**N**	**Median (IQR) or n (%)**	**N**	**Median (IQR) or n (%)**	**N**	**Median (IQR) or n (%)**	**N**
**Demographics**								
Age (years)	53.5 (17.0)	510	60.0 (15.5)	75	52.0 (18.0)	371	59.0 (15.8)	214
Male sex	196 (38.4)	510	23.0 (30.7)	75	156 (42.0)	371	63.0 (29.4)	214
BMI (kg/m²)	34.5 (9.00)	509	35.1 (10.0)	75	34.0 (9.05)	371	35.3 (9.10)	213
**Metabolic**								
Diabetes (type 1 and 2)	217 (42.5)	510	51 (68.0)	75	135 (36.4)	371	133 (62.1)	214
Hypertension	284 (55.7)	510	50 (66.7)	75	194 (52.3)	371	140 (65.4)	214
**Blood**								
AST (U/L)	36.0 (26.0)	510	45.0 (30.5)	75	33.0 (23.0)	371	45.0 (32.0)	214
ALT (U/L)	50.0 (43.8)	510	40.0 (31.0)	75	48.0 (41.0)	371	48.5 (44.8)	214
AAR	0.768 (0.302)	510	1.03 (0.475)	75	0.73 (0.306)	371	0.872 (0.34)	214
Platelets count (G/L)	234 (92.5)	510	162 (82.0)	75	243 (96.0)	371	201 (91.5)	214
HDL (mmol/L)	1.11 (0.388)	508	1.11 (0.362)	73	1.11 (0.388)	369	1.11 (0.388)	212
LDL (mmol/L)	2.64 (1.29)	497	2.22 (1.25)	71	2.66 (1.24)	361	2.46 (1.29)	207
Albumin (g/L)	44.0 (4.00)	508	43.0 (5.50)	75	44.0 (5.00)	369	43.0 (4.75)	214
GGT (UI/L)	40.0 (47.0)	507	86.5 (124)	74	37.0 (36.0)	369	63.0 (85.2)	212
Triglycerides (mmol/L)	1.64 (1.17)	508	1.54 (0.89)	73	1.55 (0.98)	369	1.74 (1.30)	212
Fasting glucose (mmol/L)	5.77 (1.80)	507	6.49 (3.03)	75	5.72 (1.55)	368	6.36 (2.37)	214
**Non-invasive tests**								
FIB-4	1.21 (0.841)	510	2.69 (2.24)	75	1.02 (0.720)	371	1.78 (1.39)	214
LSM by VCTE (kPa)	7.80 (5.30)	510	20.40 (14.3)	75	6.80 (4.35)	371	14.0 (11.3)	214
**Fibrosis stage**								
NASH CRN scoring system		510		75		371		214
F0	121 (23.7)		-		121 (32.6)		-	
F1	134 (26.3)		-		134 (36.1)		-	
F2	116 (22.7)		-		116 (31.3)		-	
F3	139 (27.3)		-		-		139 (65)	
F4	-		75 (100)		-		75 (35)	

Table S7: Patients' characteristics of French NAFLD cohort

	F<4		F=4		F<3		F≥3	
	**Median (IQR) or n (%)**	**N**	**Median (IQR) or n (%)**	**N**	**Median (IQR) or n (%)**	**N**	**Median (IQR) or n (%)**	**N**
**Demographics**								
Age (years)	57.1 (15.6)	909	62.5 (11.5)	133	55.1 (16.2)	642	61.4 (12.3)	400
Male sex	551 (60.6)	909	71.0 (53.4)	133	395 (61.5)	642	227 (56.8)	400
BMI (kg/m²)	31.2 (8.0)	905	29.8 (7.9)	132	31.0 (7.7)	638	31.6 (7.8)	399
**Metabolic**								
Diabetes (type 1 and 2)	411 (45.2)	909	97 (72.9)	133	226 (35.2)	642	282 (70.5)	400
Hypertension	-	-	-	-	-	-	-	-
**Blood**								
AST (U/L)	38.0 (24.0)	909	56.0 (33.0)	133	35.0 (19.0)	642	50.0 (27.5)	400
ALT (U/L)	56.0 (47.0)	909	58.0 (37.0)	133	55.0 (45.0)	642	59.0 (44.6)	400
AAR	0.700 (0.350)	909	0.910 (0.620)	133	0.670 (0.320)	642	0.840 (0.430)	400
Platelets count (G/L)	223 (84.0)	909	174 (88.0)	133	226.5 (82.0)	642	203 (85.0)	400
HDL (mmol/L)	1.138 (0.379)	873	1.118 (0.407)	124	1.168 (0.379)	612	1.108 (0.369)	385
LDL (mmol/L)	3.10 (1.44)	837	2.80 (1.47)	117	3.24 (1.33)	586	2.76 (1.50)	365
Albumin (g/L)	43.0 (4.8)	901	41.7 (5.3)	132	43.6 (5.0)	636	42.0 (4.6)	397
GGT (UI/L)	72.0 (98.0)	909	141.0 (231.0)	133	66.0 (92.5)	642	99.0 (124.2)	400
Triglycerides (mmol/L)	1.53 (1.05)	878	1.46 (0.87)	124	1.50 (0.97)	618	1.57 (1.03)	384
Fasting glucose (mmol/L)	5.80 (2.10)	885	6.65 (3.15)	126	5.60 (1.70)	623	6.40 (2.90)	388
**Non-invasive tests**								
FIB-4	1.31 (0.98)	909	2.55 (2.18)		1.14 (0.77)	642	1.99 (1.38)	400
LSM by VCTE (kPa)	7.80 (5.40)	909	21.3 (23.5)	133	6.70 (3.90)	642	12.6 (12.1)	400
**Fibrosis stage**								
NASH CRN scoring system		909		133		642		400
F0	116 (12.8)		-		116 (18.1)		-	
F1	240 (26.4)		-		240 (37.4)		-	
F2	286 (31.4)		-		286 (44.5)		-	
F3	267 (29.4)		-		-		267 (66.8)	
F4	-		133 (100.0)		-		133 (33.2)	

Table S8: List of predictors selected during the backward stepwise selection on a multivariable logistic regression for Agile 4 (score construction step 1).

Parameter	Estimate	Standard Error	Z	*p*-values
(Intercept)	-1.53676	0.69064	-2.22511	0.02607
LSM	0.12355	0.01079	11.44585	<0.0001
AGE	0.01975	0.0089	2.21852	0.02652
AAR	0.91865	0.24717	3.71675	0.0002
PLT	-0.01634	0.00154	-10.5791	<0.0001
TRIG	-0.2262	0.11078	-2.04193	0.04116
SEX	-0.61571	0.18346	-3.35615	0.00079
DIAB	0.72261	0.18518	3.9021	0.0001

LSM: liver stiffness; AAR: AST:ALT ratio; AST: aspartate aminotransferase; ALT: alanine aminotransferase; PLT: platelet; TRIG: triglycerides; DIAB: diabetes status.

Table S9: List of predictors selected during the backward stepwise selection on a multivariable logistic regression for Agile 3+ (score construction step 1).

Parameter	Estimate	Standard Error	Z	*p*-values
(Intercept)	-4.80695	1.13487	-4.23568	0.00002
LSM	0.24394	0.01724	14.14583	<0.0001
AGE	0.03372	0.00749	4.50306	0.00001
ALB	0.03228	0.02005	1.60985	0.10743
AAR	1.25035	0.27242	4.58976	<0.0001
PLT	-0.00907	0.00122	-7.45825	<0.0001
LDL	-0.18206	0.08135	-2.23799	0.02522
SEX	-0.37034	0.1609	-2.30167	0.02135
DIAB	0.79185	0.15403	5.14076	<0.0001
AHT	0.23289	0.15644	1.48869	0.13657

LSM: liver stiffness; ALB: albumin; AAR: AST:ALT ratio; AST: aspartate aminotransferase; ALT: alanine aminotransferase; PLT: platelet; LDL: low-density lipoproteins; DIAB: diabetes status; AHT: arterial hypertension.

Table S10: Details of predictors' removal procedure (score construction step 2) using likelihood ratio test selection procedure on nested models for Agile 4.

Removed variables	Deviance	LRT P-values	Adj. LRT P-values
Age	-5.04	0.0248	0.0289
TRIG	-4.36	0.0367	0.0367
DIAB	-15.8	7.18e-05	1.68e-04
AGE,TRIG	-9.85	0.00727	0.0102
AGE,DIAB	-23.7	7.09e-06	2.78e-05
TRIG,DIAB	-18.2	1.12e-04	1.96e-04
AGE,TRIG,DIAB	-26.4	7.95e-06	2.78e-05

TRIG: triglycerides; DIAB: diabetes status; LRT: likelihood ratio test; Adj.: adjusted. Note that for this step of score construction the aim was to decrease the number of variables included in the model to simplify its use with the following a priori assumptions: keep LSM, sex and FIB-4 components as all are readily available at time of VCTE procedure, except for age which has been shown to be linked to a decrease in performances of FIB-4 in patients 65 years of age old or older[4]. At the end of this step, triglycerides and age were removed from the predictors of Agile 4.

Table S11: Details of predictors' removal procedure (score construction step 2) using likelihood ratio test selection procedure on nested models for Agile 3+.

Removed variables	Deviance	LRT P-values	Adj. LRT P-values
AGE	-28.1122117	<0.001	<0.001
ALB	-5.7453895	0.017	0.020
LDL	-3.4650793	0.063	0.067
DIAB	-23.7081946	<0.001	<0.001
AHT	-0.5499118	0.458	0.458
AGE, ALB	-32.5257856	<0.001	<0.001
AGE, LDL	-35.6524340	<0.001	<0.001
AGE, DIAB	-59.2931137	<0.001	<0.001
AGE, AHT	-32.8680722	<0.001	<0.001
ALB, LDL	-9.4455398	0.009	0.011
ALB, DIAB	-28.8694456	<0.001	<0.001
ALB, AHT	-6.3134905	0.043	0.047
LDL, DIAB	-29.9629013	<0.001	<0.001
LDL, AHT	-4.0360988	0.133	0.137
DIAB, AHT	-25.8923807	<0.001	<0.001
AGE, ALB, LDL	-40.2126815	<0.001	<0.001
AGE, ALB, DIAB	-62.8752749	<0.001	<0.001
AGE, ALB, AHT	-37.1926983	<0.001	<0.001
AGE, LDL, DIAB	-72.0737029	<0.001	<0.001
AGE, LDL, AHT	-40.9593754	<0.001	<0.001
AGE, DIAB, AHT	-70.2726483	<0.001	<0.001
ALB, LDL, DIAB	-35.4561815	<0.001	<0.001
ALB, LDL, AHT	-10.0319000	0.018	0.021
ALB, DIAB, AHT	-31.0337739	<0.001	<0.001
LDL, DIAB, AHT	-32.4627695	<0.001	<0.001
AGE, ALB, LDL, DIAB	-75.8295091	<0.001	<0.001
AGE, ALB, LDL, AHT	-45.4029040	<0.001	<0.001
AGE, ALB, DIAB, AHT	-73.5777593	<0.001	<0.001
AGE, LDL, DIAB, AHT	-85.3141461	<0.001	<0.001
ALB, LDL, DIAB, AHT	-37.9315963	<0.001	<0.001
AGE, ALB, LDL, DIAB, AHT	-88.7114147	<0.001	<0.001

ALB: albumin; LDL: low-density lipoproteins; DIAB: diabetes status; AHT: arterial hypertension; LRT: likelihood ratio test; Adj.: adjusted. Note that for this step of score construction the aim was to decrease the number of variables included in the model to simplify its use with the following a priori assumptions: keep LSM, sex and FIB-4 components as all are readily available at time of VCTE procedure, except for age which has been shown to be linked to a decrease in performances of FIB-4 in patients 65 years of age or older[4]. At the end of this step, albumin, LDL and hypertension were removed from the predictors of Agile 3+.

Table S12: Diagnostic performances of FIB-4, liver stiffness measurement (LSM) and Agile 4 for the diagnosis of F4 in the training set, the internal validation set, the NASH CRN and the French NAFLD cohorts using a rule-in cut-off values with a 99% specificity

	**Training set**			**Internal validation set**			**NASH CRN cohort**			**French NAFLD cohort****		
	**FIB-4**	**LSM**	**Agile 4**	**FIB-4**	**LSM**	**Agile 4**	**FIB-4**	**LSM**	**Agile 4**	**FIB-4**	**LSM**	**Agile 4**
Rule out cut-off (≥85% Se)	*<1.39*	*<12.1*	*<0.251*	*<1.39*	*<12.1*	*<0.251*	*<1.39*	*<12.1*	*<0.251*	*<1.39*	*<12.1*	*<0.251*
**% patients**	50%	58%	67%	49%	59%	68%	57%	70%	77%	49%	72%	81%
**Se/Sp**	0.85/0.60	0.86/0.71	0.85/0.82	0.87/0.61	0.79/0.71	0.79/0.83	0.85/0.63	0.88/0.79	0.87/0.86	0.85/0.54	0.76/0.79	0.71/0.88
**NPV**	0.96*	0.97*	0.97*	0.97*	0.96*	0.96*	0.97	0.98	0.98	0.96	0.96	0.96
**LR-**	0.25	0.20	0.18	0.21	0.29	0.26	0.23	0.15	0.15	0.28	0.30	0.32
Indeterminate zone [85%Se ; 99%Sp[												
**% patients**	47%	39%	28%	48%	38%	27%	41%	27%	21%	48%	25%	16%
Rule in cut-off (≥99% Sp)	*≥4.94*	*≥45.5*	*≥0.843*	*≥4.94*	*≥45.5*	*≥0.843*	*≥4.94*	*≥45.5*	*≥0.843*	*≥4.94*	*≥45.5*	*≥0.843*
**% patients**	3%	3%	6%	3%	2%	4%	2%	2%	2%	3%	3%	3%
**Se/Sp**	0.11/0.99	0.11/0.99	0.21/0.99	0.10/0.99	0.09/1	0.18/1	0.11/0.99	0.15/0.99	0.17/1	0.17/0.99	0.17/0.99	0.18/0.99
**PPV**	0.64*	0.64*	0.75*	0.61*	0.78*	0.94*	0.62	0.79	1	0.65	0.81	0.80
**LR+**	12.14	11.81	20.58	10.38	24.32	97.27	10.88	24.93	Inf	12.53	30.07	27.34

Se: sensitivity; Sp: specificity. * Due to the high prevalence of cirrhosis and advanced fibrosis in the training set and the internal validation set, PPV and NPV for these datasets were adjusted on the prevalence of external validation, i·e· F4 = 13% and F≥3 = 37%. No adjustment was done for the calculation of PPV and NPV on the NASH CRN and the French NAFLD cohorts. ** Analysis performed by Pr Boursier and his team.

Table S13: Diagnostic performances of FIB-4 and liver stiffness measurement (LSM) cohorts using published cut-off values [4,5] versus Agile 3+ for the diagnosis of advanced fibrosis in the training set, the internal validation set, the NASH CRN and the French NAFLD cohorts

	Training set			Internal validation set			NASH CRN cohort			French NAFLD cohort**		
	**FIB-4**	**LSM**	**Agile 3+**	**FIB-4**	**LSM**	**Agile 3+**	**FIB-4**	**LSM**	**Agile 3+**	**FIB-4**	**LSM**	**Agile 3+**
Rule out cut-off	<1·3 (<65 y) <2·0 (≥65 y)	<8·0	<0·451	<1·3 (<65 y) <2·0 (≥65 y)	<8·0	<0·451	<1·3 (<65 y) <2·0 (≥65 y)	<8·0	<0·451	<1·3 (<65 y) <2·0 (≥65 y)	<8·0	<0·451
**% patients**	51%	32%	44%	52%	33%	42%	57%	45%	54%	52%	46%	53%
**Se/Sp**	0·71/0·77	0·91/0·57	0·85/0·78	0·71/0·78	0·90/0·61	0·87/0·76	0·68/0·71	0·85/0·63	0·82/0·75	0·70/0·67	0.87/0.66	0·83/0·75
**NPV**	0·82*	0·91*	0·90*	0·82*	0·91*	0·91*	0·79	0·88	0·88	0.78	0.89	0·87
**LR-**	0·37	0·17	0·19	0·38	0·16	0·17	0·46	0·25	0·24	0·45	0.21	0·23
Indeterminate zone												
**% patients**	32%	26%	13%	32%	26%	17%	31%	25%	16%	34%	26%	18%
Rule in cut-off	>2·67	>12·0	≥0·679	>2·67	>12·0	≥0·679	>2·67	>12·0	≥0·679	>2·67	>12·0	≥0·679
**% patients**	17%	42%	43%	16%	41%	42%	12%	30%	30%	14%	28%	29%
**Se/Sp**	0·29/0·97	0·68/0·87	0·71/0·90	0·29/0·98	0·64/0·87	0·69/0·91	0·26/0·95	0·60/0·88	0·61/0·87	0.29/0.95	0.54/0.87	0·61/0·90
**PPV**	0·85*	0·75*	0·81*	0·90*	0·73*	0·81*	0·77	0·74	0·73	0.79	0.75	0·79
**LR+**	9·97	5·15	7·16	15·30	4·63	7·33	5·71	4·97	4·70	5.94	4.75	6·20

Se: sensitivity; Sp: specificity. * Due to the high prevalence of cirrhosis and advanced fibrosis in the training set and the internal validation set, PPV and NPV for these datasets were adjusted on the prevalence of external validation, i·e· F4 = 13% and F≥3 = 37%. No adjustment was done for the calculation of PPV and NPV on the NASH CRN and the French NAFLD cohorts.

Table S14: Comparison of AUROCs of Agile 4 and Agile 3+ for patients with BMI<30kg/m² vs BMI≥30kg/m² in the internal validation set, the NASH CRN and the French NAFLD cohorts

	Internal validation set (N=646)			NASH CRN cohort (N=584)			French NAFLD cohort (N=1037)		
	**BMI<30 kg/m²**	**BMI≥30 kg/m²**		**BMI<30 kg/m²**	**BMI≥30 kg/m²**		**BMI<30 kg/m²**	**BMI≥30 kg/m²**	
**n (%)**	249 (39%)	397 (61%)		133 (23%)	451 (77%)		440 (42%)	597 (58%)	
**Fibrosis stages n(%)**									
**F0**	50 (20%)	39 (10%)		47 (35%)	74 (16%)		56 (13%)	59 (10%)	
**F1**	50 (20%)	70 (18%)		28 (21%)	106 (24%)		116 (26%)	123 (20%)	
**F2**	28 (11%)	52 (13%)		24 (18%)	92 (20%)		105 (24%)	179 (30%)	
**F3**	67 (27%)	139 (35%)		19 (14%)	119 (26%)		94 (21%)	173 (29%)	
**F4**	54 (22%)	97 (24%)		15 (11%)	60 (13%)		69 (16%)	64 (11%)	
	**AUROC [95%CI] BMI<30 kg/m²**	**AUROC [95%CI] BMI≥30 kg/m²**	**Delong test p-value**	**AUROC [95%CI] BMI<30 kg/m²**	**AUROC [95%CI] BMI≥30 kg/m²**	**Delong test p-value**	**AUROC [95%CI] BMI<30 kg/m²**	**AUROC [95%CI] BMI≥30 kg/m²**	**Delong test p-value**
**Agile 4**	0·88 [0·84;0·93]	0·91 [0·88;0·94]	0·279	0·96 [0·91;1·00]	0·93 [0·89;0·96]	0·337	0·89 [0·85;0·94]	0·89 [0·86;0·93]	0·968
**Agile 3+**	0·93 [0·90;0·96]	0·89 [0·85;0·92]	0·066	0·94 [0·90;0·98]	0·84 [0·81;0·88]	<0·001	0·90 [0·87;0·93]	0·84 [0·81;0·87]	0·005

Table S15: Comparison of AUROCs of Agile 4 and Agile 3+ for patients with steatosis severity S0/S1 vs S≥2 in the internal validation set, the NASH CRN and the French NAFLD cohorts

	Internal validation set (N=699)			NASH CRN cohort (N=585)			French NAFLD cohort (N=1033)		
	**S0/S1**	**S≥2**		**S0/S1**	**S≥2**		**S0/S1**	**S≥2**	
**n (%)**	437 (63%)	262 (37%)		258 (44%)	327 (56%)		383 (37%)	650 (63%)	
**Fibrosis stages n(%)**									
**F0**	55 (13%)	42 (16%)		61 (24%)	60 (18%)		66 (17%)	47 (7%)	
**F1**	46 (11%)	84 (32%)		52 (20%)	82 (25%)		117 (31%)	123 (19%)	
**F2**	50 (11%)	43 (16%)		45 (17%)	71 (22%)		79 (21%)	206 (32%)	
**F3**	144 (33%)	70 (27%)		56 (22%)	83 (25%)		73 (19%)	190 (29%)	
**F4**	142 (32%)	23 (9%)		44 (17%)	31 (10%)		48 (12%)	84 (13%)	
	**AUROC [95%CI] S0/S1**	**AUROC [95%CI] S≥2**	**Delong test p-value**	**AUROC [95%CI] S0/S1**	**AUROC [95%CI] S≥2**	**Delong test p-value**	**AUROC [95%CI] S0/S1**	**AUROC [95%CI] S≥2**	**Delong test p-value**
**Agile 4**	0.87 [0.84; 0.91]	0.90 [0.84; 0.95]	0.438	0.93 [0.89; 0.97]	0.94 [0.90; 0.97]	0.903	0.92 [0.83;0.91]	0.87 [0.83;0.91]	0.069
**Agile 3+**	0.90 [0.87; 0.93]	0.87 [0.83; 0.92]	0.266	0.89 [0.85; 0.93]	0.85 [0.80; 0.89]	0.176	0.89 [0.86;0.92]	0.86 [0.83;0.89]	0.142

Table S16: Comparison of AUROCs of Agile 4 and Agile 3+ for diabetic vs non-diabetic patients in the internal validation set, the NASH CRN and the French NAFLD cohorts

	Internal validation set (N=700)			NASH CRN cohort (N=585)			French NAFLD cohort (N=1042)		
	**Diabetic**	**Non-diabetic**		**Diabetic**	**Non-diabetic**		**Diabetic**	**Non-diabetic**	
**n (%)**	357 (51%)	343 (49%)		268 (46%)	317 (54%)		508 (48.8%)	534 (51.2%)	
**Fibrosis stages n(%)**									
**F0**	21 (6%)	76 (22%)		24 (9%)	97 (31%)		23 (5%)	93 (17%)	
**F1**	60 (17%)	70 (20%)		56 (21%)	78 (25%)		76 (15%)	164 (31%)	
**F2**	31 (9%)	62 (18%)		55 (20%)	61 (19%)		127 (25%)	159 (30%)	
**F3**	140 (39%)	75 (22%)		82 (31%)	57 (18%)		185 (36%)	82 (15%)	
**F4**	105 (29%)	60 (18%)		51 (19%)	24 (7%)		97 (19%)	36 (7%)	
	**AUROC [95%CI] Diabetic**	**AUROC [95%CI] Non-diabetic**	**Delong test p-value**	**AUROC [95%CI] Diabetic**	**AUROC [95%CI] Non-diabetic**	**Delong test p-value**	**AUROC [95%CI] Diabetic**	**AUROC [95%CI] Non-diabetic**	**Delong test p-value**
**Agile 4**	0.88 [0.84; 0.92]	0.90 [0.86; 0.94]	0.542	0.92 [0.88; 0.97]	0.94 [0.90; 0.98]	0.611	0.82 [0.77;0.87]	0.96 [0.94;0.98]	<0.001
**Agile 3+**	0.89 [0.85; 0.92]	0.90 [0.86; 0.93]	0.701	0.82 [0.77; 0.87]	0.88 [0.83; 0.92]	0.0946	0.80 [0.76;0.84]	0.89 [0.86;0.92]	<0.001

Table S17: Comparison of AUROCs of Agile 4 and Agile 3+ for patients with LSM measured with M probe vs XL probe in the internal validation set, the NASH CRN and the French NAFLD cohorts

	Internal validation set (N=700)			NASH CRN cohort (N=585)			French NAFLD cohort (N=999)		
	**M probe**	**XL probe**		**M probe**	**XL probe**		**M probe**	**XL probe**	
**n (%)**	411 (59%)	289 (41%)		214 (37%)	371 (63%)		564 (56%)	435 (44%)	
**Fibrosis**									
**stages n(%)**	72 (18%)	25 (8%)		64 (30%)	57 (15%)		62 (11%)	48 (11%)	
**F0**	96 (23%)	34 (12%)		46 (21%)	88 (24%)		144 (26%)	87 (20%)	
**F1**	59 (14%)	34 (12%)		45 (21%)	71 (19%)		144 (26%)	128 (29%)	
**F2**	112 (27%)	103 (36%)		38 (18%)	101 (27%)		131 (23%)	130 (30%)	
**F3**	72 (18%)	93 (32%)		21 (10%)	54 (15%)		83 (14%)	42 (10%)	
**F4**									
	**AUROC [95%CI] M probe**	**AUROC [95%CI] XL probe**	**Delong test p-value**	**AUROC [95%CI] M probe**	**AUROC [95%CI] XL probe**	**Delong test p-value**	**AUROC [95%CI] M probe**	**AUROC [95%CI] XL probe**	**Delong test p-value**
**Agile 4**	0.89 [0.86; 0.93]	0.96 [0.93; 0.99]	0.728	0.88 [0.84; 0.92]	0.92 [0.88; 0.96]	0.0729	0.89 [0.86; 0.93]	0.88 [0.83; 0.93]	0.643
**Agile 3+**	0.91 [0.88; 0.93]	0.88 [0.84; 0.92]	0.289	0.91 [0.87; 0.95]	0.84 [0.80; 0.88]	0.0215	0.90 [0.87; 0.92]	0.82 [0.78; 0.86]	0.001

Table S18: Diagnostic performances of FIB-4, LSM and Agile 3+ for the diagnosis of advanced fibrosis and of FIB-4, LSM and Agile 4 the diagnosis of cirrhosis in the training, internal and external validation sets using rule-out cut-off values with a 90% sensitivity and rule-in cut-off values with a 90% specificity

		Training set			Internal VS			NASH CRN cohort			French NAFLD cohort**		
		**FIB-4**	**LSM**	**Agile**	**FIB-4**	**LSM**	**Agile**	**FIB-4**	**LSM**	**Agile**	**FIB-4**	**LSM**	**Agile**
**F4 target**	***Rule out cut-off (≥90% Se)***	*<1.26*	*<11.1*	*<0.169*	*<1.26*	*<11.1*	*<0.169*	*<1.26*	*<11.1*	*<0.169*	*<1.26*	*<11.1*	*<0.169*
	**% patients**	44%	51%	60%	43%	53%	62%	48%	66%	71%	43%	67%	75%
	**Se/Sp**	0.90/0.54	0.90/0.64	0.90/0.75	0.90/0.54	0.87/0.65	0.85/0.76	0.89/0.54	0.89/0.74	0.89/0.80	0.89/0.48	0.81/0.75	0.76/0.83
	**NPV**	0.97*	0.98*	0.98*	0.97*	0.97*	0.97*	0.97	0.98	0.98	0.97	0.96	0.96
	**LR-**	0.19	0.15	0.14	0.18	0.21	0.20	0.20	0.14	0.13	0.24	0.25	0.29
	Indeterminate zone [90%Se ; 90%Sp[												
	**% patients**	37%	28%	15%	38%	27%	14%	38%	21%	14%	41%	21%	12%
	Rule in cut-off (≥90% Sp)	*≥2.54*	*≥19.4*	*≥0.388*	*≥2.54*	*≥19.4*	*≥0.388*	*≥2.54*	*≥19.4*	*≥0.388*	*≥2.54*	*≥19.4*	*≥0.388*
	**% patients**	19%	21%	25%	19%	20%	24%	14%	13%	15%	16%	12%	13%
	**Se/Sp**	0.50/0.90	0.58/0.90	0.74/0.90	0.44/0.89	0.56/0.91	0.69/0.90	0.52/0.92	0.57/0.94	0.71/0.94	0.50/0.89	0.53/0.94	0.58/0.93
	**PPV**	0.43*	0.46*	0.53*	0.38*	0.48*	0.50*	0.49	0.57	0.63	0.41	0.56	0.55
	**LR+**	5.09	5.71	7.43	4.15	6.21	6.72	6.63	9.14	11.63	4.77	8.70	8.49
**F≥3 target**	***Rule out cut-off (≥90% Se)***	*<0.99*	*<8.10*	*<0.351*	*<0.99*	*<8.10*	*<0.351*	*<0.99*	*<8.10*	*<0.351*	*<0.99*	*<8.10*	*<0.351*
	**% patients**	29%	32%	37%	29%	33%	36%	32%	46%	48%	27%	46%	45%
	**Se/Sp**	0.90/0.50	0.90/0.58	0.90/0.69	0.89/0.51	0.90/0.61	0.92/0.69	0.93/0.46	0.85/0.63	0.86/0.68	0.94/0.40	0.86/0.66	0.90/0.67
	**NPV**	0.89*	0.91*	0.92*	0.89*	0.91*	0.94*	0.91	0.88	0.90	0.91	0.88	0.91
	**LR-**	0.20	0.16	0.14	0.22	0.16	0.12	0.16	0.24	0.20	0.16	0.21	0.15
	Indeterminate zone [90%Se ; 90%Sp[												
	**% patients**	38%	31%	20%	35%	31%	22%	40%	29%	22%	40%	31%	26%

	Rule in cut-off (≥90% Sp)	*≥1·81*	*≥13·6*	*≥0·679*	*≥1·81*	*≥13·6*	*≥0·679*	*≥1·81*	*≥13·6*	*≥0·679*	*≥1·81*	*≥13·6*	*≥0·679*
	**% patients**	33%	37%	43%	36%	36%	42%	28%	25%	30%	33%	23%	29%
	**Se/Sp**	0·53/0·90	0·61/0·90	0·71/0·90	0·57/0·90	0·57/0·90	0·69/0·91	0·50/0·84	0·53/0·91	0·61/0·87	0·56/0·81	0·48/0·92	0·61/0·90
	**PPV**	0·76*	0·78*	0·81*	0·77*	0·77*	0·81*	0·64	0·78	0·73	0·65	0·79	0·79
	**LR+**	5·29	5·91	7·16	5·56	5·71	7·33	3·11	6·12	4·70	3·04	5·86	6·19

* Due to the high prevalence of cirrhosis and advanced fibrosis in the training set and the internal validation set, PPV and NPV for these datasets were adjusted on the prevalence of external validation, i·e· F4 = 13% and F≥3 = 37%. No adjustment was done for the calculation of PPV and NPV on the NASH CRN cohort and the French NAFLD cohort. ** Analysis performed by Pr Boursier and his team. AUROC=Area under the receiver operating curve, CI=Confidence interval, FIB-4=Fibrosis-4 index, Agile=Agile 3+ and Agile 4, LR-=Negative likelihood ratio, LR+=Positive likelihood ratio, LSM=Liver stiffness measurement, NAFLD=Nonalcoholic fatty liver disease, NASH CRN=Nonalcoholic Steatohepatitis Clinical Research Network, NPV=Negative predictive value, PPV=Positive predictive value, Se=Sensitivity, Sp=Specificity, VS=Validation set

Table S19: Comparison of AUROCs of Agile 4 and Agile 3+ for patients with liver biopsies (LB) with length > 15 mm vs length ≤ 15 mm in the internal validation set, the NASH CRN and the French NAFLD cohorts.

	Internal validation set (N=668)			NASH CRN cohort (N=585)			French NAFLD cohort (N=1041)		
	LB length>15mm	LB length≤15mm		LB length>15mm	LB length≤15mm		LB length>15mm	LB length≤15mm	
**n (%)**	229 (34%)	439 (66%)		464 (79%)	121 (21%)		931 (89.4%)	110 (10.6%)	
**Fibrosis stages n(%)**									
**F0**	39 (17%)	44 (10%)		88 (19%)	33 (27%)		108 (12%)	7 (6%)	
**F1**	58 (25%)	58 (13%)		104 (22%)	30 (25%)		218 (23%)	22 (20%)	
**F2**	49 (22%)	41 (10%)		98 (21%)	18 (15%)		261 (28%)	25 (23%)	
**F3**	64 (28%)	150 (34%)		116 (25%)	23 (19%)		233 (25%)	34 (31%)	
**F4**	19 (8%)	146 (33%)		58 (13%)	17 (14%)		111 (12%)	22 (20%)	
	**AUROC [95%CI] LB length>15mm**	**AUROC [95%CI] LB length≤15mm**	**Delong test p-value**	**AUROC [95%CI] LB length>15mm**	**AUROC [95%CI] LB length≤15mm**	**Delong test p-value**	**AUROC [95%CI] LB length>15mm**	**AUROC [95%CI] LB length≤15mm**	**Delong test p-value**
**Agile 4**	0.92 [0.86; 0.98]	0.86 [0.82; 0.89]	0.0919	0.93 [0.89; 0.96]	0.96 [0.92; 1.00]	0.1968	0·89 [0·85;0·92]	0·92 [0·86;0·98]	0·339
**Agile 3+**	0.84 [0.79; 0.90]	0.91 [0.88; 0.93]	0.0424	0.85 [0.81; 0.88]	0.93 [0.89; 0.97]	0.0046	0·87 [0·84;0·89]	0·88 [0·83;0·94]	0·566

Supplementary references

1. Vickers AJ, Elkin EB. Decision curve analysis: a novel method for evaluating prediction models. *Med Decis Making*. 2006;26(6):565. doi:10.1177/0272989X06295361
2. Majumdar A, Campos S, Gurusamy K, Pinzani M, Tsochatzis EA. Defining the Minimum Acceptable Diagnostic Accuracy of Noninvasive Fibrosis Testing in Cirrhosis: A Decision Analytic Modeling Study. *Hepatology*. 2020;71(2):627-642. doi:10.1002/HEP.30846
3. Vickers AJ, Van Calster B, Steyerberg EW. Net benefit approaches to the evaluation of prediction models, molecular markers, and diagnostic tests. *BMJ*. 2016;352. doi:10.1136/BMJ.I6
4. McPherson S, Hardy T, Dufour JF, et al. Age as a Confounding Factor for the Accurate Non-Invasive Diagnosis of Advanced NAFLD Fibrosis. *Am J Gastroenterol*. 2017;112(5):740-751. doi:10.1038/ajg.2016.453
5. Papatheodoridi M, Hiriart JB, Lupsor-Platon M, et al. Refining the Baveno VI elastography criteria for the definition of compensated advanced chronic liver disease. *J Hepatol*. December 2020. doi:10.1016/j.jhep.2020.11.050
6. Steyerberg EW. *Clinical Prediction Models*. Cham: Springer International Publishing; 2019. doi:10.1007/978-3-030-16399-0
